# The long noncoding RNA ELFN1-AS1 promotes gastric cancer growth and metastasis by interacting with TAOK1 to inhibit the Hippo signaling pathway

**DOI:** 10.1038/s41420-024-02235-5

**Published:** 2024-11-11

**Authors:** Yuanhang Wang, Kuan Shen, Quan Cheng, Xinyi Zhou, Kanghui Liu, Jian Xiao, Li Hu

**Affiliations:** 1 Department of General Surgery, Yancheng Third People’s Hospital, Affiliated Yancheng Hospital, School of Medicine, Southeast University, Yancheng, Jiangsu Province China; 2 Department of General Surgery, the First Affiliated Hospital of Nanjing Medical University, Nanjing, Jiangsu Province China; 3 Department of General Surgery, Liyang People’s Hospital, Liyang Branch Hospital of Jiangsu Province Hospital, Liyang, Jiangsu Province China

**Keywords:** Oncogenes, Gastric cancer

## Abstract

Gastric cancer (GC) is a common digestive malignancy that causes numerous cancer-related deaths. Long noncoding RNAs (lncRNAs) play a crucial role in the development of various tumors, including GC. In this study, we revealed that ELFN1-AS1, a lncRNA with aberrantly high expression, contributes to the proliferation and metastasis of GC. Mechanically, ELFN1-AS1 plays an oncogenic role by binding to the protein kinase domain of thousand and one amino acid protein kinase (TAOK1), a tumor suppressor in GC, and disrupting the TAOK1-STK3 interaction, leading to decreased STK3 phosphorylation. This decrease is accompanied by attenuation of the Hippo kinase cascade, resulting in reduced YAP1 phosphorylation, a crucial effector of the Hippo signaling pathway. Subsequently, the reduced YAP1 phosphorylation promotes its nuclear translocation, thereby enhancing the expression of MYC, a downstream target of the pathway and well-known oncogene. Taken together, the ELFN1-AS1/TAOK1/STK3/YAP1 axis may promote GC progression and is a promising target for GC treatment.

## Introduction

Gastric cancer (GC), a major threat to human health, and causes numerous cancer-related deaths [1]. Although GC diagnosis and treatment have improved considerably with the development of medical equipment and technology, the prognosis of patients with GC, particularly advanced GC, remains poor. GC develops through mutations in coding and noncoding genes. Noncoding RNAs regulate malignant behaviors of tumors, such as infiltration [2, 3], metastasis [4–6], and resistance to therapy [7–9]. Long noncoding RNAs (lncRNAs), which are >200 bp long RNA molecules, are critical regulators of these processes [10]. lncRNAs involved in GC progression include SDCBP-AS1 [11], ELF3-AS1 [12], and NEAT1 [13]. Although the oncogenic role of ELFN1-AS1 has been reported in colorectal [14], esophageal [15], and lung cancers [16], its regulatory role in GC remains unclear.

Dysregulation of various signaling pathways, such as Hippo [17], MAPK [18], and mTOR [19] pathways, contributes to tumor development. Hippo signaling is a cell growth inhibitory pathway consisting of a series of conserved kinases. This pathway controls the organ size, primarily by regulating the proliferation and apoptosis of cells. Dysregulation of Hippo signaling can result in uncontrolled tissue growth and malignant transformation. Therefore, it is important to understand cancer pathogenesis [20, 21].

Thousand and one amino acid protein kinase (TAOK1) is involved in several processes, including positive regulation of the JNK cascade, negative microtubule depolymerization, and mitotic G2 DNA damage checkpoint signaling. A previous study reported that Tao-1, a homolog of TAOK1, in *Drosophila* can phosphorylate Hpo kinase, thereby regulating the Hippo-Salvador-Warts tumor suppressor pathway [22]. A similar phosphorylation event between TAOK1 and MST2/STK3, which are homologs of TAO-1 and Hpo, respectively, has been demonstrated in mammals [23].

This study revealed the presence of the lncRNA ELFN1-AS1 with aberrantly high expression in GC. ELFN1-AS1 plays an oncogenic role by binding to TAOK1 and preventing STK3 phosphorylation by TAOK1, thereby inhibiting the Hippo signaling pathway and promoting GC progression.

## Results

### Upregulation of ELFN1-AS1 expression in GC is associated with poor prognosis

**Fig. 1:**
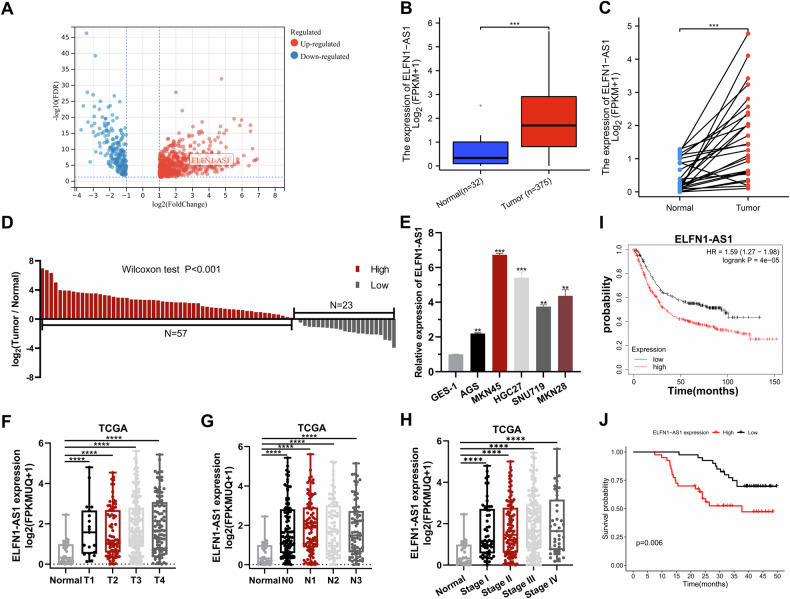
TCGA database analysis of ELFN1-AS1 expression levels, clinical relevance, and ELFN1-AS1 expression levels in tissue samples and gastric cancer cell lines. **A** TCGA-STAD dataset differential expression analysis showing differentially expressed genes. **B** Expression status of ELFN1-AS1 in unpaired samples from TCGA-STAD dataset. **C** Expression status of ELFN1-AS1 in paired samples from TCGA-STAD dataset. **D** qRT-PCR detection of ELFN1-AS1 in 80 pairs of gastric cancer tissues and paired precancerous non-tumor tissues. **E** Quantitative PCR assay of ELFN1-AS1 expression levels in five gastric cancer cell lines and normal gastric mucosal epithelial cells (GES-1). **F**–**H** Correlation of ELFN1-AS1 expression levels with TNM stage. **I** Correlation of ELFN1-AS1 expression levels with prognosis. **J** Survival analysis of 80 pairs of gastric cancer patients in our center according to the expression level of ELFN1-AS1. *p < 0.05, **p < 0.01, ***p < 0.001, ****p < 0.0001.

We analyzed RNA sequencing data from the TCGA-STAD dataset to identify lncRNAs involved in GC pathogenesis. Our analysis revealed that ELFN1-AS1 expression was considerably upregulated in GC cells (Fig. 1A–C). This finding was validated by examining ELFN1-AS1 expression in 80 pairs of GC tissues and paraneoplastic nontumor tissues collected at our center (Fig. 1D). Furthermore, ELFN1-AS1 expression was significantly upregulated in various GC cell lines (Fig. 1E). MKN45 and AGS cells were selected for subsequent assays. We investigated the clinical relevance of ELFN1-AS1 expression in the TCGA database and revealed that high ELFN1-AS1 expression was observed in patients with more advanced TNM stages (Fig. 1F–H). High ELFN1-AS1 expression indicated larger tumor size, deeper tumor infiltration, more advanced TNM stage, more pronounced lymph node metastasis, and lower tumor differentiation in the GC cohort (Table S1). Notably, Kaplan–Meier analysis revealed that high expression of ELFN1-AS1 indicated poor prognosis (http://www.kmplot.com) (Fig. 1I); a similar result was observed in our cohort (Fig. 1J). Analysis of the coding ability of ELFN1-AS1 using the Coding-Potential Assessment Tool revealed that ELFN1-AS1 lacks protein-coding ability (Fig. S1).

### ELFN1-AS1 promotes the proliferation, migration, and invasion of GC cells

**Fig. 2:**
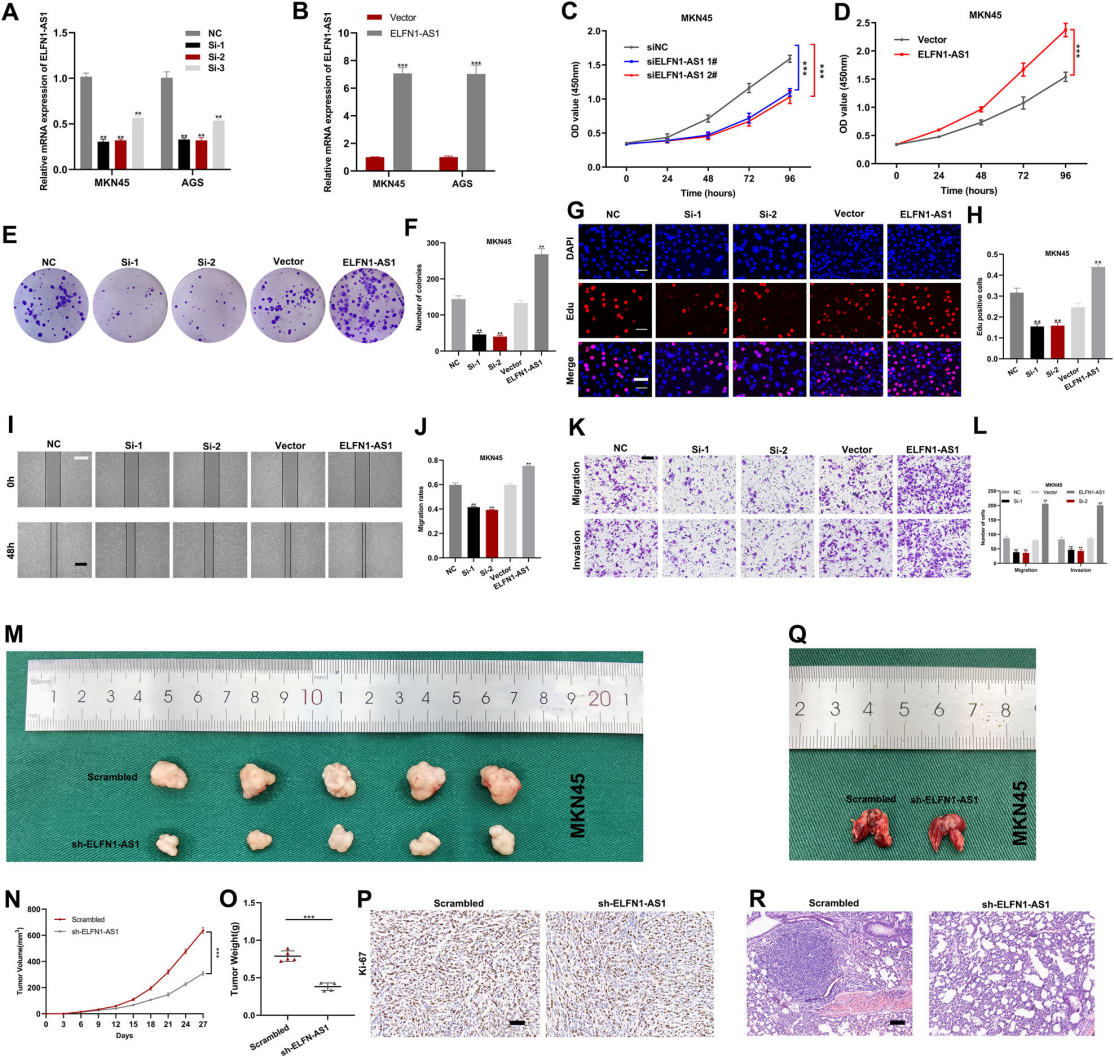
ELFN1-AS1 promotes the proliferation, migration, invasion, and metastasis of GC. **A**, **B** Detection of knockdown and overexpression efficiency in MKN45 with qRT-PCR. **C**, **D** The growth curves detected by CCK-8 assay after knockdown or overexpression of ELFN1-AS1 in MKN45. **E**, **F** Cloning formation assay was applied to evaluate the effect of knockdown of ELFN1-AS1 on the proliferation of MKN45. **G**, **H** The proliferative capacity of MKN45 was evaluated by EdU assay (scale bar: 100 μm). **I**, **J** Wound healing assay in MKN45 with knockdown or overexpression of ELFN1-AS1 to evaluate migration ability (scale bar: 100 μm). **K**, **L** Transwell assays were used to detect migration and invasion ability in MKN45 with knockdown or overexpression of ELFN1-AS1(scale bar: 200 μm). **M** Xenograft tumors of ELFN1-AS1 knockdown or control group. **N**, **O** Tumor size and weight of different groups. **P** Ki-67 expression was detected by immunohistochemical staining (scale bar: 50 μm). **Q**, **R** Representative images of lung metastasis and HE staining of the specimen (scale bar: 100 μm). **p < 0.01, ***p < 0.001.

We transfected GC cells with siRNA targeting ELFN1-AS1 or ELFN1-AS1 overexpression plasmids and evaluated knockdown or overexpression efficiency (Fig. 2A, B). Then, we examined the effect of altered ELFN1-AS1 expression on cell proliferation. *ELFN1-AS1* knockdown significantly decreased and *ELFN1-AS1* overexpression increased the proliferation of cells, as observed in the CCK-8 assay (Fig. 2C, D and Fig. S2A, B), colony formation assay (Fig. 2E, F and Fig. S2C, D), and EdU assay (Fig. 2G, H and Fig. S2E, F). *ELFN1-AS1* knockdown suppressed the migration and invasion of GC cells, whereas *ELFN1-AS1* overexpression promoted it, as demonstrated by wound healing and Transwell assays (Fig. 2I–L and Fig. S2G–J). These results confirm the role of ELFN1-AS1 in GC malignancy.

### ELFN1-AS1 knockdown inhibits tumor growth and metastasis

To investigate the effect of altered ELFN1-AS1 expression on tumorigenesis and metastasis in vivo, we constructed a subcutaneous transplantation tumor model and lung metastasis model in nude mice with MKN45 cells. The mean volume and weight of xenograft tumors decreased significantly after *ELFN1-AS1* knockdown (Fig. 2M–O). Immunohistochemical analysis of xenograft tumors revealed a significant decrease in Ki-67 expression after *ELFN1-AS1* knockdown (Fig. 2P). Further, we observed that lung metastasis decreased after *ELFN1-AS1* knockdown, and hematoxylin-eosin (HE) staining of lung tissue revealed a similar result (Fig. 2Q, R).

### ELFN1-AS1 binds to TAOK1 and does not affect TAOK1 expression

**Fig. 3:**
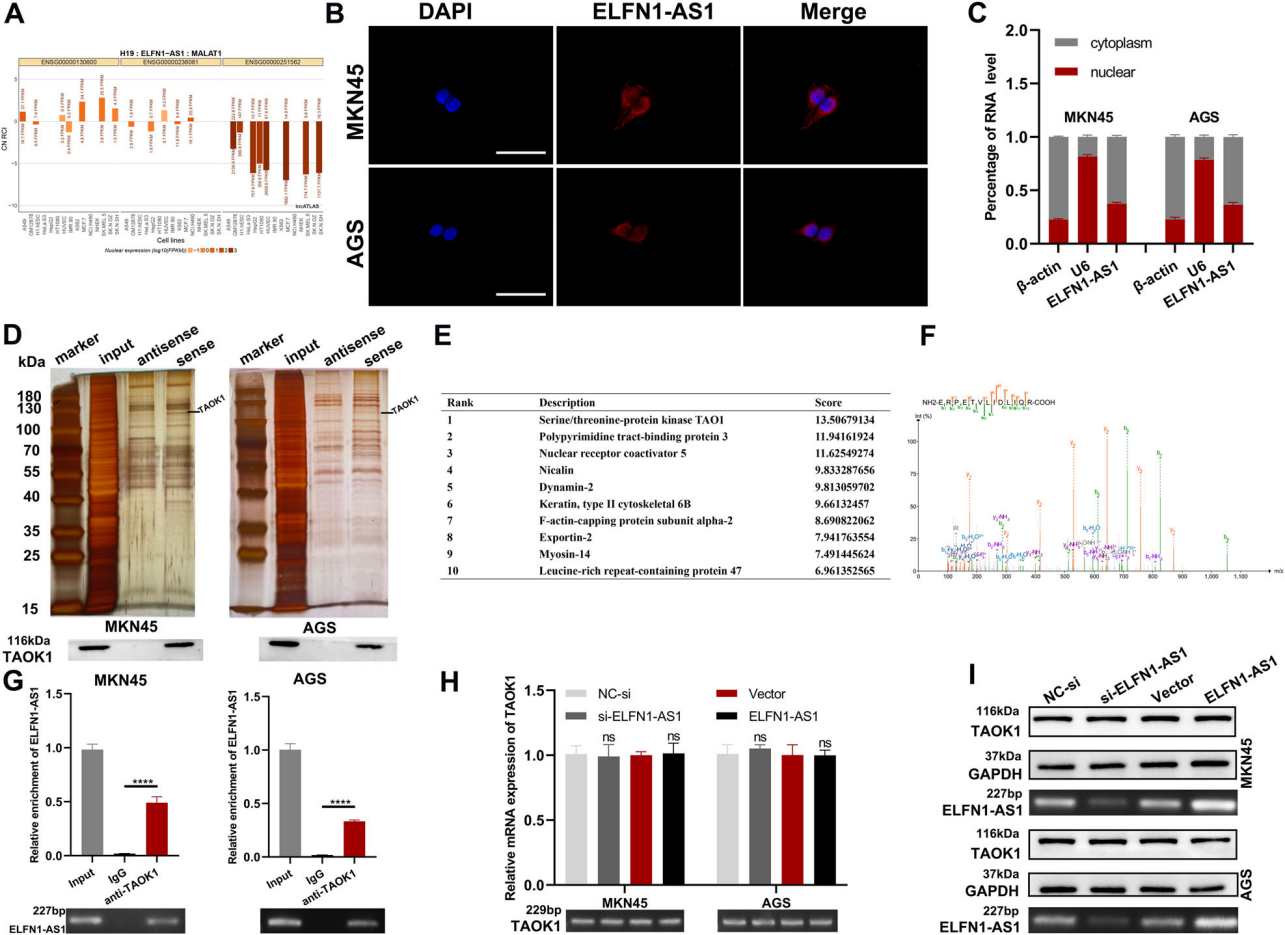
ELFN1-AS1 localizes in the cytoplasm, binds to TAOK1, and does not affect TAOK1 expression. **A** lncALTAS predicted ELFN1-AS1 was mainly localized in the cytoplasm. **B, C** Subcellular localization of ELFN1-AS1 was detected by FISH assay and qRT-PCR in MKN45 and AGS (scale bar: 50 μm). **D** RNA pulldown assay and silver staining assay were applied to find ELFN1-AS1 binding protein, and the result was validated by western blot. **E** The top 10 proteins that bind to ELFN1-AS1. **F** The secondary mass spectrometry of TAOK1 protein. **G** RIP assay showed that ELFN1-AS1 could be enriched by TAOK1 in cell lysates of MKN45 and AGS. **H**, **I** ELFN1-AS1 did not affect TAOK1 expression at either the mRNA or protein levels.

lncATLAS (http://lncatlas.crg.eu/) predicted that ELFN1-AS1 is mainly located in the cytoplasm (Fig. 3A). FISH and subcellular fractionation assays confirmed the predicted outcome (Fig. 3B, C). Competitive endogenous RNAs (ceRNAs) are widely used to explore cytoplasmically localized lncRNAs, and studies have suggested that ELFN1-AS1 acts as a ceRNA. In light of this, we considered whether ELFN1-AS1 might function in other ways. Therefore, we performed the RNA pulldown assay to identify proteins that bind to ELFN1-AS1. Silver staining revealed specific protein bands in the ELFN1-AS1 probe group (sense group) compared with the control group (antisense group) (Fig. 3D). Mass spectrometry analysis showed that among the top 10 proteins that specifically bind to ELFN1-AS1, TAOK1 showed the highest binding capacity (Fig. 3E, F). Further, TAOK1 was detectable in input and sense groups, but not in the antisense group (Fig. 3D). Compared with IgG, the RIP assay showed significant enrichment of ELFN1-AS1 by TAOK1 (Fig. 3G), indicating specific binding between ELFN1-AS1 and TAOK1. Thus, *ELFN1-AS1* knockdown or overexpression did not significantly alter TAOK1 expression at the mRNA or protein level (Fig. 3H, I).

### TAOK1 inhibits the proliferation, migration, invasion, and metastasis of GC cells

**Fig. 4:**
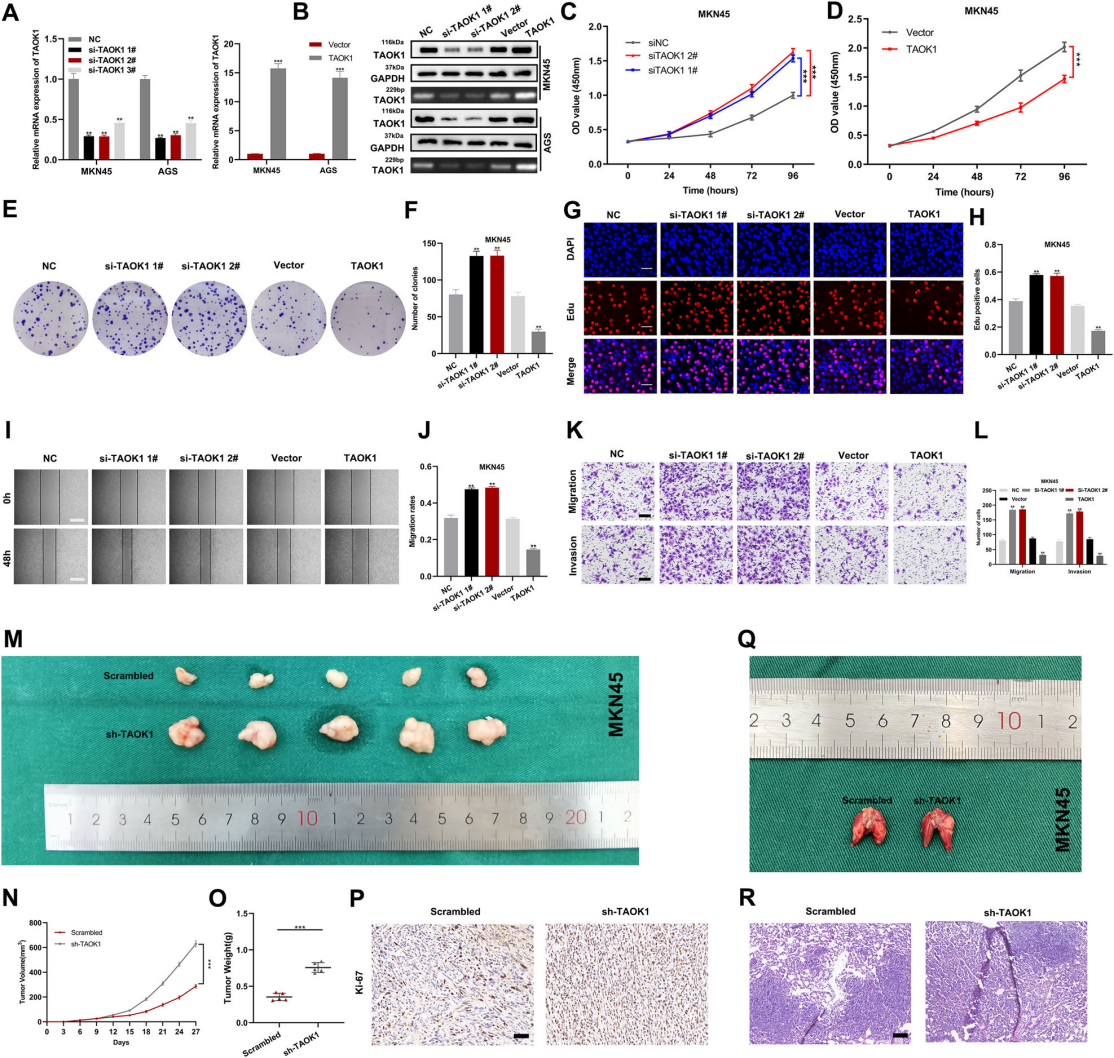
TAOK1 inhibits the proliferation, migration, invasion, and metastasis of GC. **A**, **B** Detection of knockdown and overexpression efficiency in MKN45 and AGS with qRT-PCR and western blot. **C**, **D** The growth curves detected by CCK-8 assay after knockdown or overexpression of TAOK1 in MKN45. **E**, **F** Cloning formation assay was applied to evaluate the effect of knockdown of TAOK1 on the proliferation of MKN45. **G**, **H** The proliferative capacity of MKN45 was evaluated by EdU assay (scale bar: 100 μm). **I**, **J** Wound healing assay in MKN45 with knockdown or overexpression of TAOK1 to evaluate migration ability (scale bar: 100 μm). **K**, **L** Transwell assays were used to detect migration and invasion ability in MKN45 with knockdown or overexpression of TAOK1(scale bar: 200 μm). **M** Xenograft tumors of TAOK1 knockdown or control group. **N**, **O** Tumor size and weight of different groups. **P** Ki-67 expression was detected by immunohistochemical staining (scale bar: 50 μm). **Q**, **R** Representative images of lung metastasis and HE staining of the specimen (scale bar: 100 μm). **p < 0.01, ***p < 0.001.

To evaluate the role of TAOK1 in GC, we transfected GC cells with sh-*TAOK1* and TAOK1 overexpression plasmids and detected the silencing and overexpression efficiency (Fig. 4A, B). We evaluated the proliferative capacity of cells with altered TAOK1 expression. CCK-8, EdU, and colony formation assays revealed that *TAOK1* knockdown reduced the proliferative capacity of GC cells, whereas *TAOK1* overexpression showed the opposite effect (Fig. 4C–H and Fig. S3A–F). Transwell and wound healing assays further validated the results that *TAOK1* knockdown facilitated migration and invasion of GC cells, whereas *TAOK1* overexpression showed opposite effects (Fig. 4I–L and Fig. S3G–J). Thus, the results confirmed that TAOK1 is a tumor suppressor in vitro.

### TAOK1 inhibits GC tumor growth and metastasis

*TAOK1* knockdown significantly increased the growth rate and average weight of tumors (Fig. 4M–O). Immunohistochemical staining of xenograft tumors revealed a considerable increase in Ki-67 expression after *TAOK1* knockdown (Fig. 4P). The lung metastasis model in nude mice showed a significant increase in lung metastasis after *TAOK1* knockdown (Fig. 4Q). HE staining of lung tissue confirmed that *TAOK1* knockdown significantly increased the cross-sectional area of local metastasis (Fig. 4R). These findings revealed that TAOK1 suppresses tumor formation and metastasis in vivo.

### TAOK1 mediates the effect of ELFN1-AS1 on GC

**Fig. 5:**
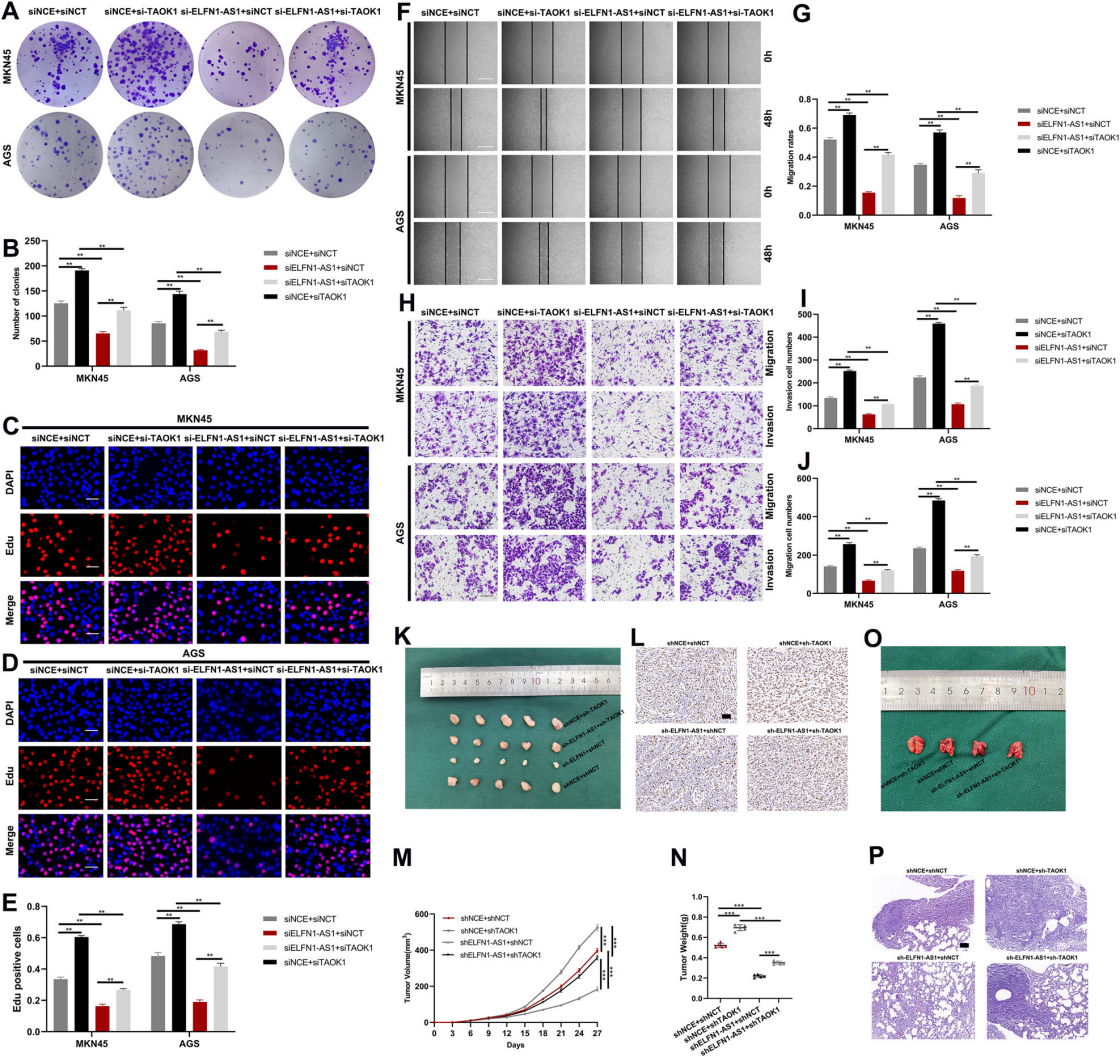
ELFN1-AS1 exerts biological functions by TAOK1. **A**, **B** Colony formation assay suggested that co-transfection of si-ELFN1-AS1 and si-TAOK1 counteracted the suppressing effects caused by ELFN1-AS1 knockdown. **C**–**E** EdU assay revealed that the suppressing effects caused by ELFN1-AS1 knockdown could be rescued by TAOK1 knockdown (scale bar: 100 μm). **F, G**. Wound healing assay showed that the knockdown of TAOK1 restored the suppressive effect of ELFN1-AS1 knockdown on cell migration (scale bar: 100 μm). **H**–**J** Transwell assays showed that the knockdown of TAOK1 rescued the suppressive effect of ELFN1-AS1 knockdown on cell migration and invasion (scale bar: 200 μm). **K** Xenograft tumors of different groups. **L** Ki-67 expression was detected by immunohistochemical staining in different groups (scale bar: 50 μm). **M**, **N** Tumor size and weight of different groups. **O**, **P** Representative images of lung metastasis and HE staining of the specimen (scale bar: 100 μm). **p < 0.01, ***p < 0.001.

To examine the ELFN1-AS1–TAOK1 interaction, we cotransfected GC cells with sh-*TAOK1* and sh-*ELFN1-AS1*. EdU, colony formation, Transwell, and wound healing assays showed that *TAOK1* knockdown partially rescued the effect of ELFN1-AS1 slicing on GC cells (Fig. 5A–J). The subcutaneous xenograft tumor formation assay and lung metastasis assay after cotransfecting GC cells with si-*ELFN1-AS1* and si-*TAOK1* showed that *TAOK1* knockdown partially rescued the effect of ELFN1-AS1 slicing on tumor formation and metastasis (Fig. 5K–P). Thus, TAOK1 mediates the effect of ELFN1-AS1 on GC.

### ELFN1-AS1 inhibits STK3 phosphorylation by TAOK1 and suppresses Hippo signaling pathway activation

**Fig. 6:**
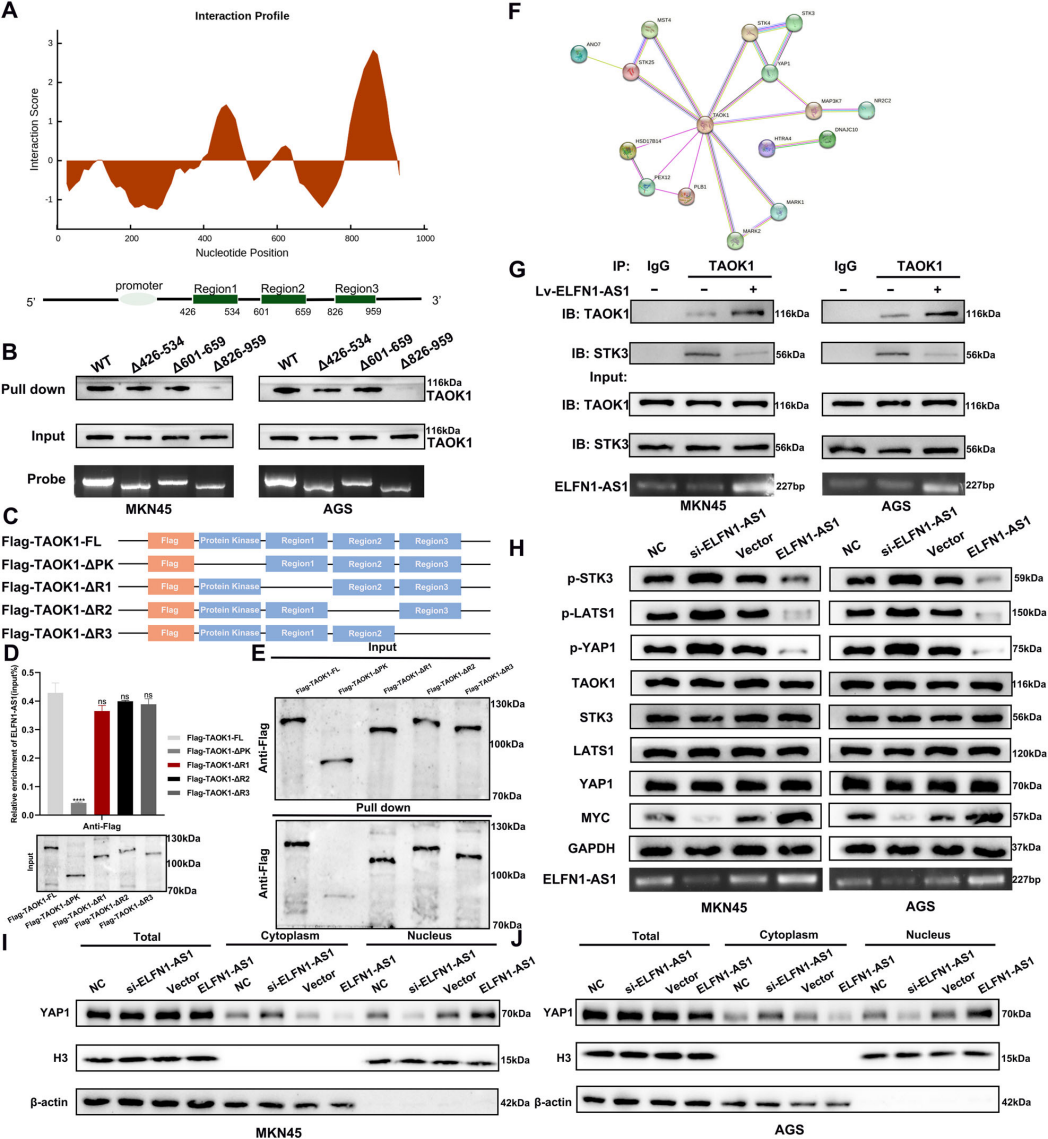
ELFN1-AS1 inhibits the phosphorylation of STK3 by TAOK1, thereby inhibiting Hippo pathway activation. **A** CatRAPID predicted possible fragments of ELFN1-AS1 binding to TAOK1. **B** RNA pulldown assay was used to detect the binding ability of ELFN1-AS1 after deletion of the corresponding fragment compared to full-length ELFN1-AS1 to TAOK1. **C**, **D** The full-length or truncated forms of flag-labeled recombinant TAOK1 protein were incubated with HEK293T cell lysates, and then the enrichment of ELFN1-AS1 was detected by qRT-PCR. **E** RNA pulldown assays were conducted using ELFN1-AS1-specific probes against full-length or truncated Flag-tagged recombinant TAOK1 protein forms. **F** Bioinformatics predicted the protein network interacting with TAOK1. **G** COIP assay was used to verify the interaction between TAOK1 and STK3 and the effect of overexpression of ELFN1-AS1 on the interaction between them. **H** Effect of altered ELFN1-AS1 expression on the expression levels of various effector molecules in the Hippo signaling pathway. **I**, **J** Effect of altered ELFN1-AS1 expression on the level of YAP1 distribution in the nucleus and cytoplasm of cells. ****p < 0.0001.

As TAOK1 exerts carcinostatic effects and its expression is unaffected by ELFN1-AS1, we examined the ELFN1-AS1–TAOK1 interaction. First, we utilized CatRAPID tool (http://service.tartaglialab.com) to predict the fragments of ELFN1-AS1 that bind to TAOK1 and identified three potential fragments (426–534, 601–659, and 826–959 bp) (Fig. 6A). Then, we performed RNA pulldown experiments using biotin-labeled probes with truncated ELFN1-AS1 mutants. Western blot results showed that compared with the full-length ELFN1-AS1 probe, probes with 426–534-bp and 601–659-bp deletions could bind to TAOK1, whereas those with 826–959-bp deletion showed a significantly reduced ability to bind to TAOK1 (Fig. 6B). Thus, the 826–959 bp fragment of ELFN1-AS1 is crucial for the binding of ELFN1-AS1 to TAOK1. We then explored the domain of TAOK1 that binds to ELFN1-AS1. UniProt (https://www.uniprot.org/) analysis showed that TAOK1 has four domains (Fig. 6C). To determine the structural domain of TAOK1 that binds to ELFN1-AS1, we established TAOK1 mutants with truncated protein structural domains. The RIP assay in HEK293T cells revealed that the protein kinase (PK) domain of TAOK1 specifically binds to ELFN1-AS1 (Fig. 6D). In addition, the RNA pulldown assay in HEK293T cells demonstrated that TAOK1 with truncated PK domain could not be pulled down by ELFN1-AS1 probes (Fig. 6E). Thus, the results suggested that ELFN1-AS1 binds to the PK domain of TAOK1.

**Fig. 7:**
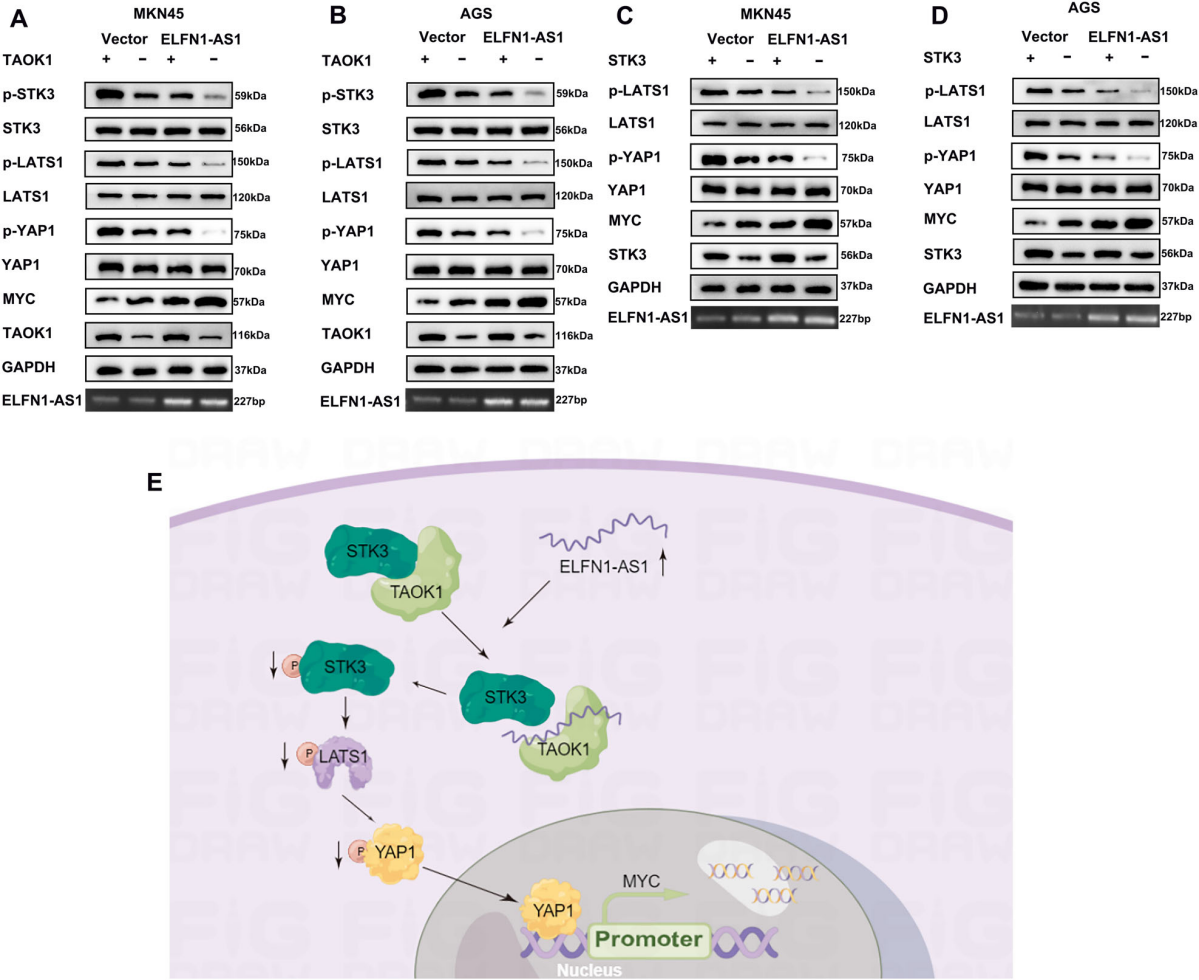
Interaction of ELFN1-AS1 with TAOK1 acts through the Hippo signaling pathway. **A**, **B** Overexpression of TAOK1 rescued the effect of ELFN1-AS1 overexpression on the expression of essential proteins of the Hippo signaling pathway. **C**, **D** Overexpression of STK3 rescued the effect of ELFN1-AS1 overexpression on the expression of essential proteins of the Hippo signaling pathway. **E** Model describing the role of ELFN1-AS1 as an oncogene by inhibiting the Hippo signaling pathway in GC.

We investigated whether the binding of ELFN1-AS1 to the PK domain of TAOK1 affects its kinase activity. Studies have shown that TAOK1 phosphorylates STK3 [22–24]. Protein interaction network analysis revealed that STK3 is an interacting protein of TAOK1 (https://cn.string-db.org/) (Fig. 6F). In addition, immunoprecipitation results supported the interaction between TAOK1 and STK3, and *ELFN1-AS1* overexpression significantly inhibited their interaction (Fig. 6G). Subsequently, we demonstrated that *ELFN1-AS1* knockdown significantly increased STK3, LATS1, and YAP1 phosphorylation and dramatically decreased MYC expression; ELFN1-AS1 overexpression displayed opposite effects (Fig. 6H). However, the expression of STK3, LATS1, and YAP1 was unaffected by the altered expression of ELFN1-AS1 (Fig. 6H). *ELFN1-AS1* knockdown led to increased distribution of YAP1 in the cytoplasm, whereas its overexpression caused increased distribution of YAP1 in the nucleus, consistent with the changes in YAP1 phosphorylation level (Fig. 6I, J). Furthermore, *ELFN1-AS1* overexpression-mediated inhibition of the Hippo signaling pathway could be partially reversed by *TAOK1* or *STK3* overexpression (Fig. 7A–D). Taken together, ELFN1-AS1 inhibits TAOK1-mediated STK3 phosphorylation by binding to the PK domain of TAOK1. This further inhibits LATS1 and YAP1 phosphorylation, thereby facilitating the entry of YAP1 into the nucleus for cotranscriptional activation (Fig. 7E).

## Discussion

Although the role of lncRNAs in various cancers has been extensively studied [25, 26], their role in GC remains unknown. In this study, we identified ELFN1-AS1 as a novel lncRNA associated with GC. Consistently, a study reported that ELFN1-AS1 promotes GC progression [27]. We confirmed that the high expression of ELFN1-AS1 is related to poor prognosis in GC.

Studies have revealed the mechanisms through which lncRNAs mediate their effects [28, 29], and one of the most extensively investigated mechanisms is ceRNA [30]. In addition, the interaction between lncRNAs and RNA-binding proteins is crucial [31]. lncRNAs can bind to transcription factors and recruit them to gene promoter regions [32]. lncRNAs can also bind to methyltransferases or transcriptional repressors, such as EZH2, and recruit them to gene promoter regions to enhance histone methylation, thereby leading to transcriptional enhancement or repression depending on the type of histone methylation [33, 34]. lncRNAs can directly modulate protein expression by binding to proteins, which inhibits their binding to ubiquitin ligases, thereby inhibiting protein degradation through the ubiquitination pathway [17]. lncRNAs have been reported to regulate mRNA splicing, transport, degradation, and translation by interacting with proteins. Some studies have suggested that lncRNAs can promote or inhibit protein modification after binding to proteins [35, 36]. Our study identified ELFN1-AS1 as a novel binding partner of TAOK1. We demonstrated the tumor suppressive effects of TAOK1 and showed that the oncogenic effects of ELFN1-AS1 were achieved to some extent by TAOK1. We revealed that the 826–959 bp fragment of ELFN1-AS1 is essential for its binding to TAOK1 and that ELFN1-AS1 binds to the PK domain of TAOK1. Furthermore, ELFN1-AS1 binding to TAOK1 inhibits the interaction of TAOK1 with STK3, thus reducing STK3 phosphorylation.

The activation of STK3, an important kinase in the Hippo signaling pathway, promotes LATS1 and LATS2 activation, which subsequently phosphorylates YAP/TAZ, allowing them to remain in the cytoplasm [21, 37, 38]. We revealed that ELFN1-AS1 expression was negatively correlated with STK3, LATS1, and YAP1 phosphorylation, suggesting that ELFN1-AS1 inhibits the Hippo signaling pathway. Under these conditions, YAP/TAZ translocates to the nucleus and acts as a cotranscriptional activator by binding to TEAD and promoting gene transcription [38]. Our results demonstrated that *ELFN1-AS1* overexpression increased YAP1 accumulation in the nucleus, whereas *ELFN1-AS1* knockdown showed the opposite effect. In addition, MYC expression was negatively correlated with *ELFN1-AS1* overexpression. These results suggest that ELFN1-AS1 inhibits the Hippo signaling pathway by binding to TAOK1, thus promoting GC progression.

Our study has several limitations. The interactions between different cytokines during tumor development remain unclear. ELFN1-AS1 may promote GC through other signaling pathways or interaction with other proteins. TAOK1 has been implicated in regulating the P38 MAPK pathway. Thus, the potential effect of ELFN1-AS1 binding to TAOK1 on this pathway warrants further investigation [39, 40]. Additionally, according to a previous study, MYC could promote *ELFN1-AS1* expression as a transcription factor in colorectal cancer [41]. We demonstrated that ELFN1-AS1 inhibited the Hippo signaling pathway, leading to the nuclear translocation of YAP1 and subsequent cotranscriptional activation, thereby promoting MYC expression. Therefore, studies must investigate the presence of a positive feedback loop between ELFN1-AS1 and MYC in GC.

In conclusion, we identified ELFN1-AS1, a highly expressed lncRNA in GC that is related to poor prognosis. ELFN1-AS1 inhibits TAOK1-mediated STK3 phosphorylation by binding to the PK domain of TAOK1, further inhibiting the kinase phosphorylation cascade and suppressing the Hippo signaling pathway to promote GC progression. Our study provides insights into GC development and suggests that the ELFN1-AS1/TAOK1/STK3/YAP1 axis is a promising target for GC therapy.

## Materials and methods

### Human tissue collection

GC and paired paraneoplastic tissues were collected from 80 patients who underwent radical GC surgery without neoadjuvant radiotherapy before surgery at The First Affiliated Hospital of Nanjing Medical University (NMU). Tissues were frozen in liquid nitrogen and stored at -80°C immediately after resection. Written informed consent was obtained before specimen collection. This study was approved by the Medical Ethics Committee of the First Affiliated Hospital of Nanjing Medical University.

### Cell lines and cell transfection experiments

The cell lines GES-1, MKN28, AGS, SNU719, HGC27, and MKN45 were purchased from the Chinese Academy of Sciences (Shanghai, China). All cells were cultured in RMPI-1640 medium (WISENT, Nanjing, China) except AGS, which was cultured in F12K medium (WISENT, Nanjing, China). All cells were incubated at 37°C under 5%CO_2_. All media contained 10% fetal bovine serum (Gibco, New York, USA) and 1% antibiotics (100 U/mL penicillin and 100 mg/mL streptomycin). The authenticity of cells was tested using short tandem repeat analysis.

GC cells were transfected with nucleotide sequences using Lipofectamine 3000 (Invitrogen, USA). Full-length TAOK1, STK3, and ELFN1-AS1 were subcloned into an overexpression plasmid obtained from Generay Biotech (Shanghai, China). Small interfering RNAs (siRNAs) targeting ELFN1-AS1 and TAOK1 were synthesized by Generay Biotech. GenePharma (Shanghai, China) synthesized sh-ELFN1-AS1 and sh-TAOK1. Related sequences are listed in Table S2.

### Cell counting, colony formation, and 5-ethynyl-2′-deoxyuridine assays

The cell counting kit-8 (CCK8), colony formation, and 5′ -ethynyl-2′-deoxyuridine (Edu) assays were used to evaluate the proliferation of GC cells, according to a previous study [30].

### Wound healing and transwell assays

Both assays were performed to evaluate the migration and invasion of GC cells, as described previously [30].

### RNA extraction and quantitative reverse transcription polymerase chain reaction (qRT–PCR)

Total RNA was extracted using a TRIzol reagent (Invitrogen, Carlsbad, CA, USA). Complementary DNA was synthesized using Prime Script RT Reagent Kit (Monad, Wuhan, China) was applied to qRT-PCR and data analysis were conducted as described in a previous study [42]. Primers used in the experiment are listed in Table S3.

### RNA fluorescent in situ hybridization (FISH) and subcellular fractionation assay

FISH was performed using a FISH kit (RiboBio, Guangzhou, China). The Cy3-labeled ELFN1-AS1 probe was synthesized by RiboBio (Guangzhou, China). Images were acquired using the Thunder Imager Fast High-Resolution Inverted Fluorescence Imaging System (LEICA, Wetzlar, Germany). RNA isolation was performed using a PARIS Kit (Invitrogen, Carlsbad, CA, USA). Gene expression in different cell fractions was analyzed via qRT-PCR.

### RNA pulldown, mass spectrometry, and silver staining

Biotin-labeled probes or antisense sequences and three deletion constructs, with 426–534, 601-659, and 826–959 bp, were synthesized by BersinBio (Guangzhou, China). The assay was conducted using an RNA pulldown Kit (BersinBio, Guangzhou, China). Protein bands were visualized via silver staining and further analyzed using mass spectrometry. The unique proteins of both groups were scored with Percolator [43] and listed in Table S5 and Table S6.

### RNA-protein immunoprecipitation (RIP) and coimmunoprecipitation (CoIP)

The RIP assay was conducted using an RNA Immunoprecipitation Kit (BersinBio, Guangzhou, China). Briefly, MKN45, AGS, and HEK293T cell lysates were incubated overnight with antibodies (3 μg), followed by 1 h incubation with protein A/G beads. RNA enrichment was evaluated via qRT-PCR. The CoIP assay was performed using a Co-Immunoprecipitation Kit (BersinBio, Guangzhou, China). Proteins were detected via western blot. Related antibodies are listed in Table S4.

### Immunohistochemistry analysis and hematoxylin-eosin staining

Staining was conducted as previously described [42].

### Western blot

Western blot was performed as described previously [42]. Antibodies used in this experiment are listed in Table S4. The uncropped blots are shown in Supplementary Fig. S4 and Fig. S5.

### Animal experiments

For subcutaneous tumorigenesis in nude mice, MKN45 cells were prepared as a single-cell suspension at a density of 1 × 10^6^ cells in 100uL PBS and then injected into the upper middle groin on both sides of nude mice. Tumor volume was calculated as follows: volume (mm^3^) = 0.5 × length (mm) × width^2^ (mm^2^). Four weeks later, the mice were euthanized, and subcutaneous tumors were weighed and collected for imaging. For the lung metastasis model, MKN45 cells were prepared similarly and injected into nude mice through the tail vein. Nude mice were dissected 4 weeks later to monitor lung metastasis via imaging. All animal experiments were grouped by randomized single-blinding. Animal experiments were approved by the Committee on the Ethics of Animal Experiments of Nanjing Medical University.

### Public data analysis and statistical analysis

Gene expression data of 372 GC tissues and 35 normal tissues in TCGA-STAD (version number 07-20-2019) [30] were obtained from TCGA (https://portal.gdc.cancer.gov/). Statistical data were analyzed using SPSS 26.0 and GraphPad Prism 9.0. Experimental data are expressed as mean ± standard deviation. The significance of differences between groups was analyzed using independent *t*-test, Wilcoxon test, chi-square test, and ANOVA. Each experiment was conducted at least thrice; *p*-values of <0.05 were considered to indicate statistical significance.

## Supplementary information

Supplementary materials

## Data Availability

The authors declared that all and the other data supporting the results of this study are available within the paper and its additional files.
