# Supplementary material for: The long noncoding RNA ELFN1-AS1 promotes gastric cancer growth and metastasis by interacting with TAOK1 to inhibit the Hippo signaling pathway
Source: Cell Death Discov. 2024 Nov 11;10:465. doi: 10.1038/s41420-024-02235-5 (PMC11555383; doi:10.1038/s41420-024-02235-5)
Supplement: Supplementary file 1 — Supplementary materials [file 41420_2024_2235_MOESM1_ESM.pdf]

Figure S1

Result for species name : hg19 with job ID :1673314008							
Data ID	Sequence Name	RNA Size	ORF Size	Ficket Score	Hexamer Score	Coding Probability	Coding Label
0	NR_120508.1	1008	189	1.0321	-0.00123941157022	0.053147841289574	no

Figure S2

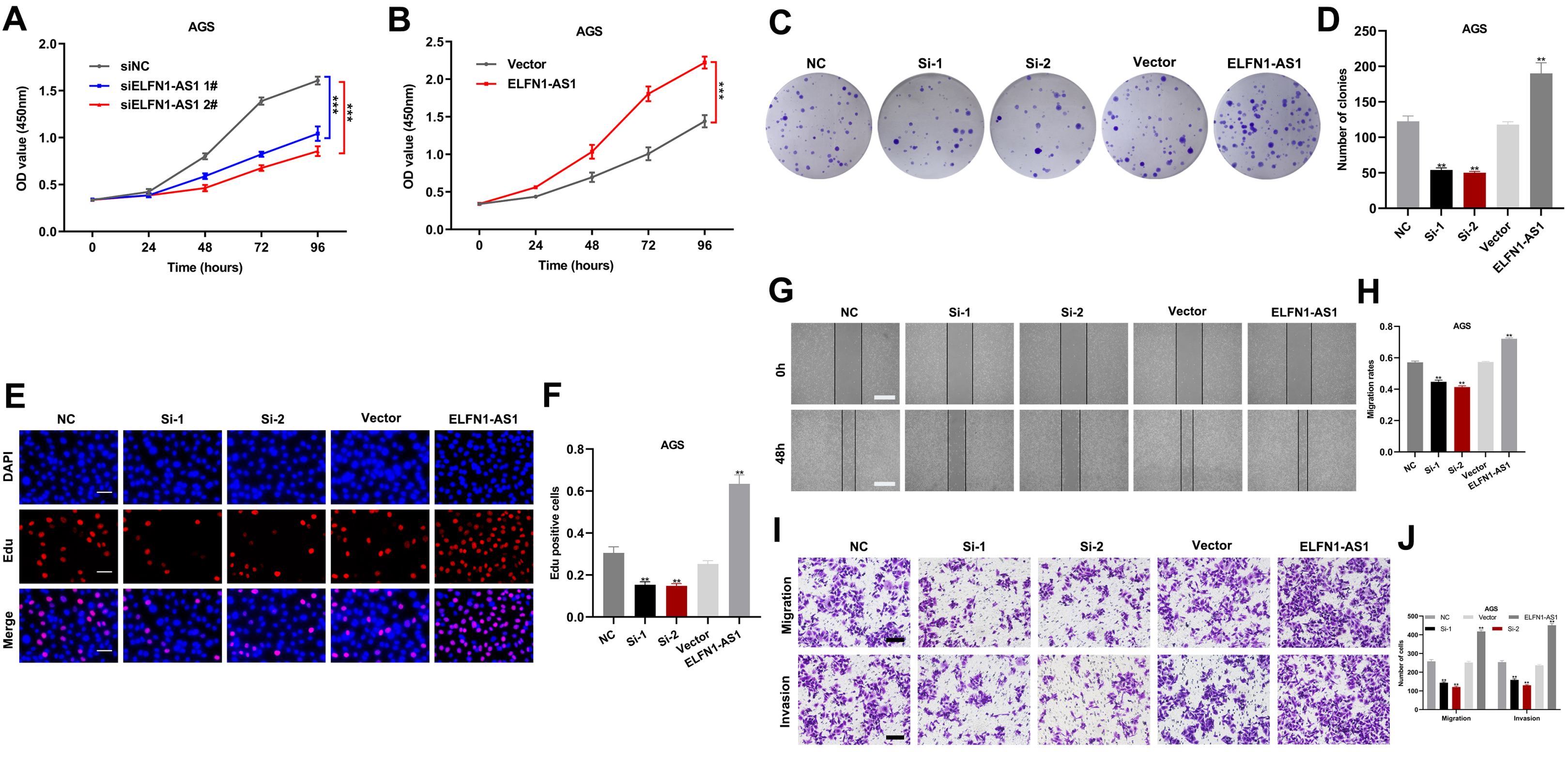
A
AGS
siNC
siELFN1-AS1 1#
siELFN1-AS1 2#
OD value (450nm)
Time (hours)
B
AGS
Vector
ELFN1-AS1
OD value (450nm)
Time (hours)
C
NC
Si-1
Si-2
Vector
ELFN1-AS1
D
AGS
Number of clonies
E
NC
Si-1
Si-2
Vector
ELFN1-AS1
DAPI
Edu
Merge
F
AGS
Edu positive cells
G
0h
48h
H
AGS
Migration rates
I
Migration
Invasion
J
AGS
Number of cells
Migration
Invasion

Figure S3

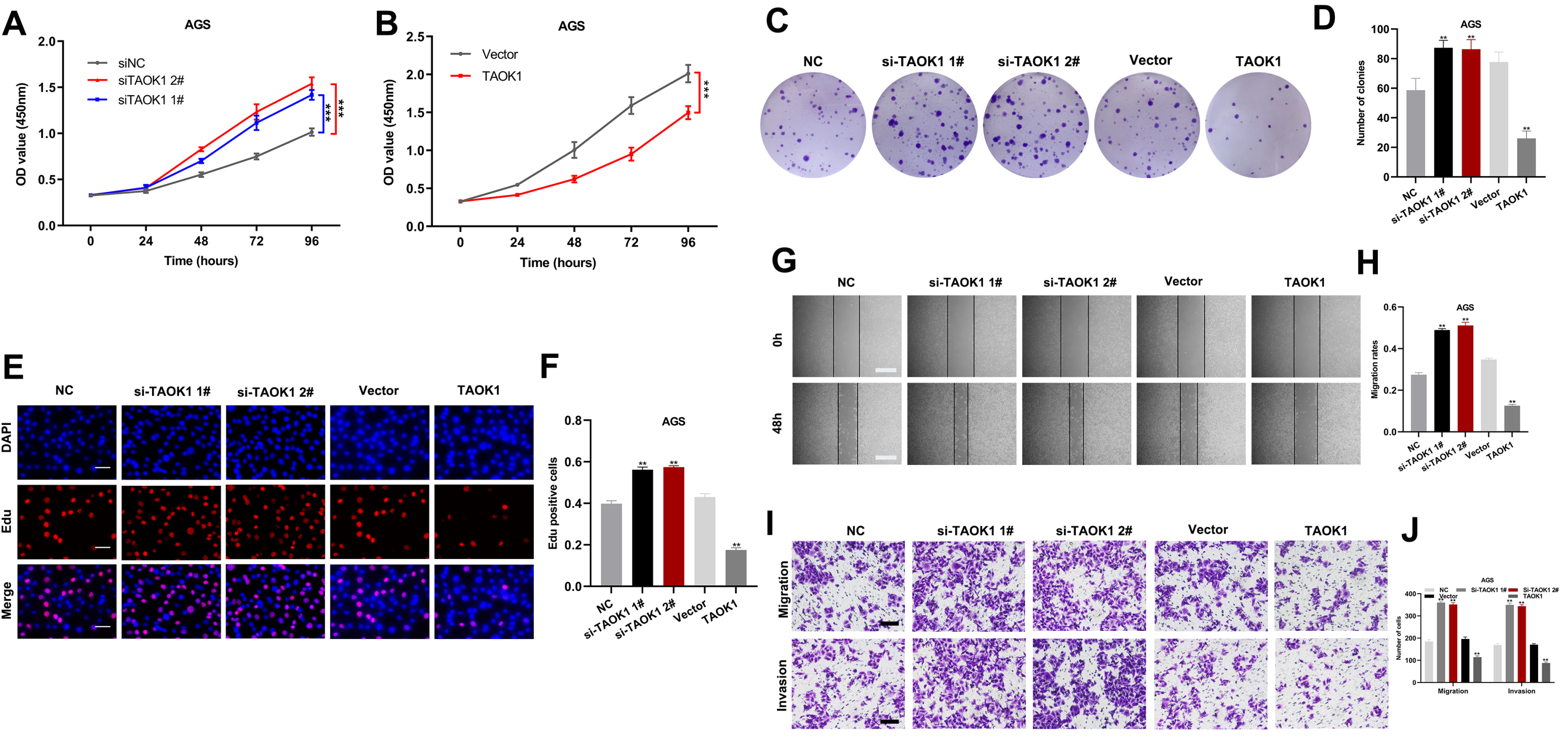

A
AGS
siNC
siTAOK1 2#
siTAOK1 1#
OD value (450nm)
Time (hours)
B
AGS
Vector
TAOK1
OD value (450nm)
Time (hours)
C
NC
si-TAOK1 1#
si-TAOK1 2#
Vector
TAOK1
D
AGS
Number of clonies
E
NC
si-TAOK1 1#
si-TAOK1 2#
Vector
TAOK1
DAPI
Edu
Merge
F
AGS
Edu positive cells
G
0h
48h
H
AGS
Migration rates
I
Migration
Invasion
J
AGS
Number of cells
Migration
Invasion

Supplementary Figure legends

Figure S1. Coding ability analysis of ELFN1-AS1 with Coding-Potential Assessment Tool (CPAT).

Figure S2. ELFN1-AS1 promotes the proliferation, migration, and invasion of GC cells. A, B. The growth curves detected by CCK-8 assay after knockdown or overexpression of ELFN1-AS1 in AGS. **C, D.** Cloning formation assay was applied to evaluate the effect of knockdown of ELFN1-AS1 on the proliferation of AGS. **E, F.** The proliferative capacity of AGS was evaluated by EdU assay (scale bar: 100μm). **G, H.** Wound healing assay in AGS with knockdown or overexpression of ELFN1-AS1 to evaluate migration ability (scale bar: 100μm). **I, J.** Transwell assays were used to detect migration and invasion ability in AGS with knockdown or overexpression of ELFN1-AS1(scale bar: 200μm). $^{**}p < 0.01$， $^{***}p < 0.001$.

Figure S3. TAOK1 inhibits the proliferation, migration, and invasion of GC cells. A, B. The growth curves detected by CCK-8 assay after knockdown or overexpression of TAOK1 in AGS.**C, D.** Cloning formation assay was applied to evaluate the effect of knockdown of TAOK1 on the proliferation of AGS. **E, F.** The proliferative capacity of AGS was evaluated by EdU assay (scale bar: 100μm). **G, H.** Wound healing assay in AGS with knockdown or overexpression of TAOK1 to evaluate migration ability (scale bar: 100μm). **I, J.** Transwell assays were used to detect migration and invasion ability in AGS with knockdown or overexpression of TAOK1(scale bar: 200μm). $^{**}p < 0.01$， $^{***}p < 0.001$.

Figure S4

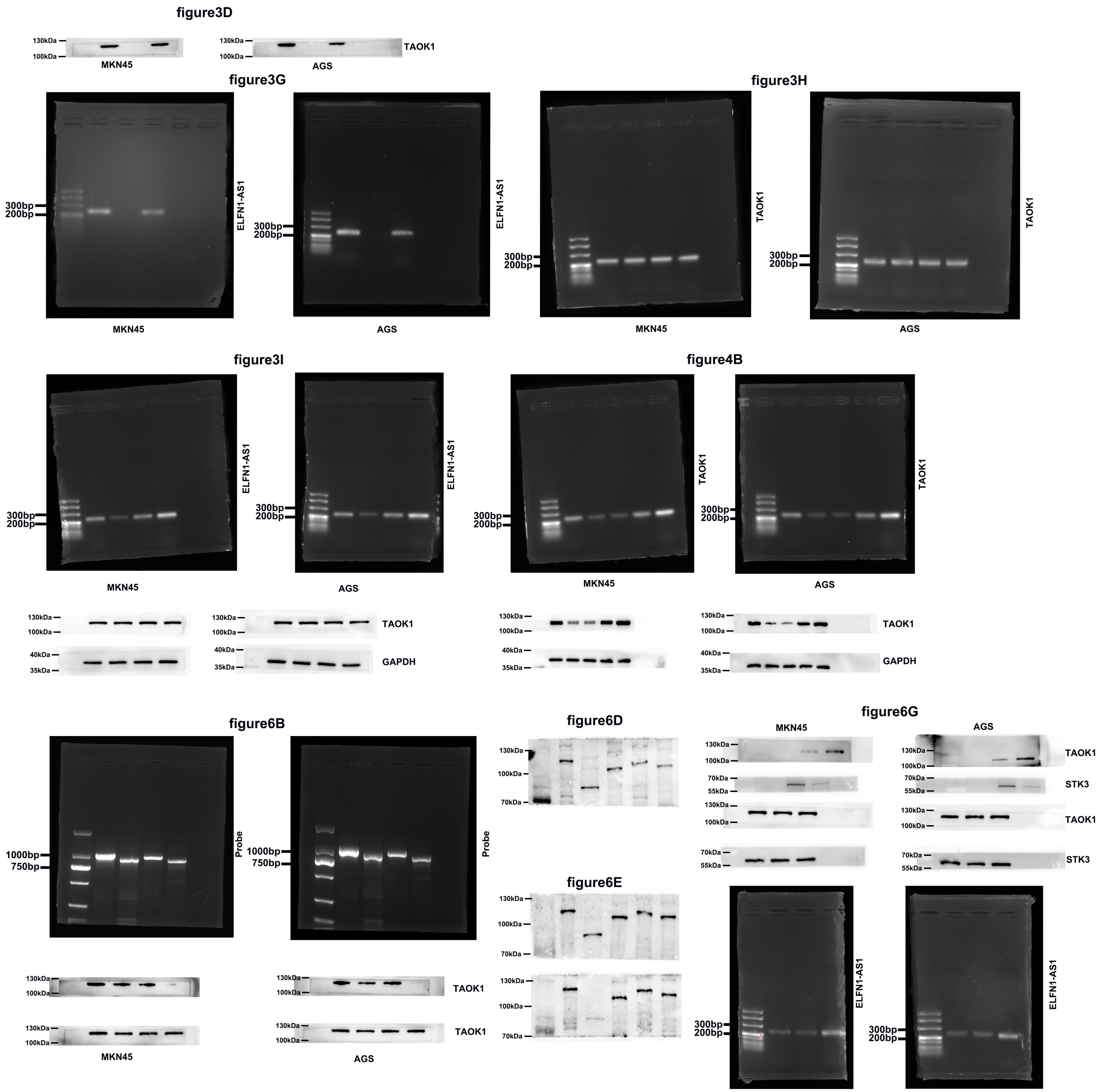
figure3D
130kDa
100kDa
MKN45
AGS
TAOK1
figure3G
300bp
200bp
ELFN1-AS1
MKN45
AGS
figure3H
TAOK1
MKN45
AGS
figure3I
ELFN1-AS1
MKN45
AGS
figure4B
TAOK1
MKN45
AGS
130kDa
100kDa
TAOK1
40kDa
35kDa
GAPDH
figure6B
1000bp
750bp
Probe
MKN45
AGS
figure6D
130kDa
100kDa
70kDa
figure6E
figure6G
MKN45
AGS
TAOK1
70kDa
55kDa
STK3
ELFN1-AS1
300bp
200bp

Figure S5

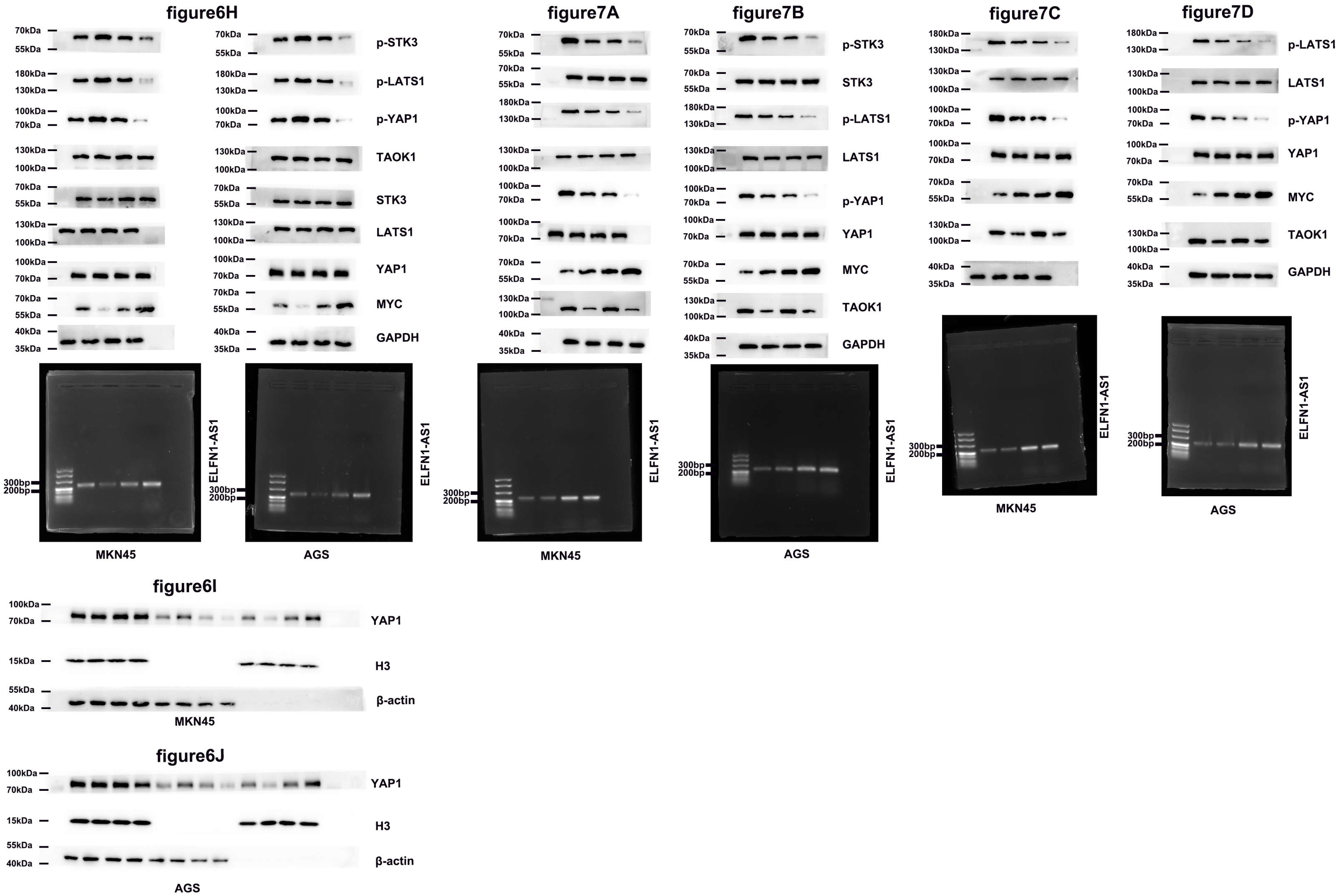
figure6H
figure7A
figure7B
figure7C
figure7D
p-STK3
p-LATS1
p-YAP1
TAOK1
STK3
LATS1
YAP1
MYC
GAPDH
ELFN1-AS1
300bp
200bp
MKN45
AGS
figure6I
YAP1
H3
β-actin
MKN45
figure6J
YAP1
H3
β-actin
AGS
180kDa
130kDa
100kDa
70kDa
55kDa
40kDa
35kDa
15kDa

Table S1. Correlation between ELFN1-AS1 expression and the clinicopathologic parameters in 80 GC patients

Characteristic	Group	Case	Expression of ELFN1-AS1		p-value
			High	Low	
Age	<60	41	18	23	
	≥60	39	22	17	0.371
Gender	Male	51	22	29	
	Female	29	18	11	0.162
Tumor size	< 4cm	26	11	25	
	≥ 4cm	54	29	15	**0.003**
Tumor site	Cardia	26	14	12	
	Non-cardia	54	26	28	0.812
Tumor differentiation	Well + moderate	26	8	18	
	Poor	54	32	22	**0.031**
T stage	T1-T2	35	11	24	
	T3-T4	45	29	16	**0.006**
Lymph node status	Negative	26	5	21	
	Positive	54	35	19	**< 0.001**
TNM stage	I–II	40	14	26	
	III–IV	40	26	14	**0.013**

$p < 0.05$ was considered statistically significant.

Table S2. siRNA and shRNA used in this study

siRNA/shRNA		5'to 3'
si-ELFN1-AS1-1	Sense	GCUUGUGGUUUCUCACAAATT
	Antisense	UUUGUGAGAAACCACAAGCTT
si-ELFN1-AS1-2	Sense	CCUUUAAUCUCUUGCUCAATT
	Antisense	UUGAGCAAGAGAUUAAAGGTT
si-ELFN1-AS1-3	Sense	AGCUUGUGGUUUCUCACAATT
	Antisense	UUGUGAGAAACCACAAGCUTT
si-TAOK1-1	Sense	GGAGAAACUUAUCAAGAAATT
	Antisense	UUUCUUGAUAAGUUUCUCCTT
si-TAOK1-2	Sense	GCACAAAGCUGUUCUGAAATT
	Antisense	UUUCAGAACAGCUUUGUGCTT
si-TAOK1-3	Sense	CGAAGAGAACGAGAACUAATT
	Antisense	UUAGUUCUCGUUCUCUUCGTT
sh-ELFN1-AS1	Sense	CACCGCATTCTGGACTCCTCTCTTTCGAAAAAGAG AGGAGTCCAGAATGC
	Antisense	AAAAGCATTCTGGACTCCTCTCTTTTTCGAAAGAG AGGAGTCCAGAATGC
sh-TAOK1	Sense	CACCGCGCCCTGAAACAGTGTTAATCGAAATTAAC ACTGTTTCAGGGCGC
	Antisense	AAAAGCGCCCTGAAACAGTGTTAATTTCGATTAAC ACTGTTTCAGGGCGC

Table S3. Primers used in the study

Gene	Primer	5' to 3'
ELFN1-AS1	Forward	CTCGCTAACCACCAAGGC
	Reverse	TCTCGGAGTGAATGACAGGA
TAOK1	Forward	TCAGGCCCAACAGAAGAAAGAA
	Reverse	ACGGCATTCCAGCTCTAGGT
STK3	Forward	TGTTTACTTCGTCCACGCCA
	Reverse	CAGCAGATTTGGAGGGGGTC
β-actin	Forward	GCATCGTCACCAACTGGGAC
	Reverse	ACCTGG CCGTCAGGCAGCTC
U6	Forward	CTCGCTTCGGCAGCACA
	Reverse	GCGAGCACAGAATTAATACGAC
GAPDH	Forward	TGCACCACCAACTGCTTAGC
	Reverse	GGCATGGACTGTGGTCATGAG

Table S4. Antibodies used in this study

Product	Source	No. of Catalogue
**Western blot**		
**Primary antibody**		
Anti-TAOK1	ZEN-BIOSCIENCE	R27334
Anti-STK3	ZEN-BIOSCIENCE	R25816
Anti-p-STK3	Proteintech	80093-1-RR
Anti-LATS1	ZEN-BIOSCIENCE	252567
Anti-p-LATS1	Cell Signaling Technology	#9157
Anti-YAP1	Proteintech	13584-1-AP
Anti-p-YAP1	ZEN-BIOSCIENCE	381297
Anti-MYC	ZEN-BIOSCIENCE	380784
Anti-GAPDH	Proteintech	60004-1-Ig
Anti-β-actin	ZEN-BIOSCIENCE	380624
Anti-IgG	Proteintech	30000-0-AP
Anti-H3	Proteintech	17168-1-AP
Anti-Flag	Proteintech	20543-1-AP
**Secondary antibody**		
anti-rabbit IgG-HRP	Proteintech	SA00001-15
anti-mouse IgG-HRP	Proteintech	SA00001-1
**IHC**		
**Primary antibody**		
Anti-Ki67	Abcam	ab15580
**Secondary antibody**		
Goat Anti-Rabbit IgG H&L (Alexa Fluor® 594)	Abcam	ab150080

Table S5. mass spectrometry data of the sense group (experimental group)

Protein_ID	Protein_Qscore
sp|Q7L7X3|TAOK1_HUMAN	13.50679134
sp|O95758|PTBP3_HUMAN	11.94161924
sp|Q9HCD5|NCOA5_HUMAN	11.62549274
sp|Q969V3|NCLN_HUMAN	9.833287656
sp|P50570|DYN2_HUMAN	9.813059702
sp|P04259|K2C6B_HUMAN	9.66132457
sp|P47755|CAZA2_HUMAN	8.690822062
sp|P55060|XPO2_HUMAN	7.941763554
sp|Q7Z406|MYH14_HUMAN	7.491445624
sp|Q8N1G4|LRC47_HUMAN	6.961352565
sp|P81605|DCD_HUMAN	6.555525104
sp|Q8WVV9|HNRLL_HUMAN	6.555525104
sp|P13646|K1C13_HUMAN	6.087429117
sp|P62424|RL7A_HUMAN	6.021659361
sp|Q9H857|NT5D2_HUMAN	6.021659361
sp|Q06210|GFPT1_HUMAN	5.91995988
sp|Q32P28|P3H1_HUMAN	5.708331488
sp|P17858|PFKAL_HUMAN	5.708331488
sp|Q92616|GCN1_HUMAN	5.67747307
sp|P21333|FLNA_HUMAN	5.585575898
sp|Q8N163|CCAR2_HUMAN	5.308861565
sp|P14625|ENPL_HUMAN	5.291538006
sp|O95793|STAU1_HUMAN	5.281499786
sp|Q86W42|THOC6_HUMAN	5.209377083
sp|P57088|TMM33_HUMAN	5.161639066
sp|A0FGR8|ESYT2_HUMAN	4.974153596
sp|P78386|KRT85_HUMAN	4.848024852
sp|A0A075B6P5|KV228_HUMAN	4.319155237
sp|P08754|GNAI3_HUMAN	4.319155237
sp|Q9NVJ2|ARL8B_HUMAN	4.319155237
sp|Q8TB52|FBX30_HUMAN	4.319155237
sp|P84243|H33_HUMAN	4.319155237
sp|Q5T200|ZC3HD_HUMAN	4.319155237
sp|O75569|PRKRA_HUMAN	3.277762552
sp|P06702|S10A9_HUMAN	3.277762552
sp|Q13393|PLD1_HUMAN	3.277762552
sp|A6NKB5|PCX2_HUMAN	3.277762552
sp|Q9UJC5|SH3L2_HUMAN	3.277762552
sp|Q9BYD3|RM04_HUMAN	3.277762552
sp|O14656|TOR1A_HUMAN	3.277762552
sp|O95347|SMC2_HUMAN	3.277762552
sp|O75676|KS6A4_HUMAN	3.277762552

sp|Q08380|LG3BP_HUMAN	3.277762552
sp|P28290|ITPI2_HUMAN	3.277762552
sp|Q13895|BYST_HUMAN	3.277762552
sp|Q04671|P_HUMAN	3.277762552
sp|Q5PRF9|SMAG2_HUMAN	3.277762552
sp|Q9Y4I1|MYO5A_HUMAN	3.277762552
sp|O95219|SNX4_HUMAN	3.277762552
sp|Q08945|SSRP1_HUMAN	3.277762552
sp|Q9UL03|INT6_HUMAN	3.277762552
sp|P40938|RFC3_HUMAN	3.277762552
sp|P62266|RS23_HUMAN	3.277762552
sp|Q2TB90|HKDC1_HUMAN	3.277762552
sp|Q8WUK0|PTPM1_HUMAN	3.277762552
sp|P07864|LDHC_HUMAN	3.277762552
sp|O43707|ACTN4_HUMAN	3.277762552
sp|Q96KA5|CLP1L_HUMAN	3.277762552
sp|Q8TAA3|PSMA8_HUMAN	3.277762552
sp|Q9BXF6|RFIP5_HUMAN	3.277762552
sp|P43003|EAA1_HUMAN	3.277762552
sp|Q86VM9|ZCH18_HUMAN	3.277762552
sp|Q9NUJ3|T11L1_HUMAN	3.277762552
sp|Q9BT22|ALG1_HUMAN	3.277762552
sp|Q15695|U2AFL_HUMAN	3.277762552
sp|O14773|TPP1_HUMAN	3.277762552
sp|Q92685|ALG3_HUMAN	3.277762552
sp|Q9NVE7|PANK4_HUMAN	3.277762552
sp|Q15003|CND2_HUMAN	3.277762552
sp|Q96KP1|EXOC2_HUMAN	3.277762552
sp|P0C0S5|H2AZ_HUMAN	3.06613416
sp|P24539|AT5F1_HUMAN	3.06613416
sp|P16298|PP2BB_HUMAN	3.06613416
sp|P26038|MOES_HUMAN	3.06613416
sp|Q96EY7|PTCD3_HUMAN	3.06613416
sp|O43747|AP1G1_HUMAN	3.06613416
sp|Q8WU17|RN139_HUMAN	3.06613416
sp|Q9UKU7|ACAD8_HUMAN	3.06613416
sp|P09622|DLDH_HUMAN	3.06613416
sp|Q16401|PSMD5_HUMAN	2.909746816
sp|P42166|LAP2A_HUMAN	2.909746816
sp|Q9Y6N5|SQOR_HUMAN	2.909746816
sp|Q5SRE5|NU188_HUMAN	2.909746816
sp|Q9BZ23|PANK2_HUMAN	2.809666565
sp|P62258|1433E_HUMAN	2.809666565
sp|O75586|MED6_HUMAN	2.809666565

sp|O43592|XPOT_HUMAN	2.809666565
sp|Q8NI29|FBX27_HUMAN	2.809666565
sp|Q8NEM2|SHCBP_HUMAN	2.809666565
sp|Q9P035|HACD3_HUMAN	2.743896809
sp|Q96TC7|RMD3_HUMAN	2.743896809
sp|O00566|MPP10_HUMAN	2.743896809
sp|Q8IVL5|P3H2_HUMAN	2.743896809
sp|Q93075|TATD2_HUMAN	2.743896809
sp|Q15477|SKIV2_HUMAN	2.743896809
sp|O95678|K2C75_HUMAN	2.667564328
sp|Q8TAA5|GRPE2_HUMAN	2.642197328
sp|P61353|RL27_HUMAN	2.642197328
sp|Q96EK4|THA11_HUMAN	2.642197328
sp|O15234|CASC3_HUMAN	2.642197328
sp|P26639|SYTC_HUMAN	2.642197328
sp|P09382|LEG1_HUMAN	2.642197328
sp|Q9Y5J1|UTP18_HUMAN	2.642197328
sp|Q15654|TRIP6_HUMAN	2.642197328
sp|P26640|SYVC_HUMAN	2.642197328
sp|Q92896|GSLG1_HUMAN	2.642197328
sp|P47897|SYQ_HUMAN	2.642197328
sp|Q13363|CTBP1_HUMAN	2.642197328
sp|Q8N122|RPTOR_HUMAN	2.642197328
sp|Q6XZF7|DNMBP_HUMAN	2.642197328
sp|Q96KR1|ZFR_HUMAN	2.642197328
sp|Q96CU9|FXRD1_HUMAN	2.600342509
sp|P11310|ACADM_HUMAN	2.600342509
sp|Q8NG99|OR7G2_HUMAN	2.600342509
sp|Q8N8D1|PDCD7_HUMAN	2.552377491
sp|Q9BYN8|RT26_HUMAN	2.519441738
sp|P52926|HMGA2_HUMAN	2.519441738
sp|O43819|SCO2_HUMAN	2.519441738
sp|P43353|AL3B1_HUMAN	2.519441738
sp|O43818|U3IP2_HUMAN	2.519441738
sp|Q96P63|SPB12_HUMAN	2.519441738
sp|P62316|SMD2_HUMAN	2.519441738
sp|P05109|S10A8_HUMAN	2.519441738
sp|Q06787|FMR1_HUMAN	2.519441738
sp|Q86YW9|MD12L_HUMAN	2.519441738
sp|P48556|PSMD8_HUMAN	2.519441738
sp|Q13185|CBX3_HUMAN	2.519441738
sp|Q9H9Y6|RPA2_HUMAN	2.519441738
sp|Q9BXC9|BBS2_HUMAN	2.519441738
sp|Q14697|GANAB_HUMAN	2.519441738

sp|Q15005|SPCS2_HUMAN	2.519441738
sp|Q14966|ZN638_HUMAN	2.519441738
sp|O15067|PUR4_HUMAN	2.519441738
sp|Q96N46|TTC14_HUMAN	2.519441738
sp|P57740|NU107_HUMAN	2.519441738
sp|P52948|NUP98_HUMAN	2.519441738
sp|Q8NB90|AFG2H_HUMAN	2.519441738
sp|Q96A65|EXOC4_HUMAN	2.519441738
sp|Q8IX01|SUGP2_HUMAN	2.519441738
sp|Q5THK1|PR14L_HUMAN	2.519441738
sp|Q9UPY3|DICER_HUMAN	2.519441738
sp|Q13724|MOGS_HUMAN	2.519441738
sp|Q9NSD9|SYFB_HUMAN	2.519441738
sp|Q9Y4H2|IRS2_HUMAN	2.487201416
sp|Q9H336|CRLD1_HUMAN	2.487201416
sp|Q8IWA0|WDR75_HUMAN	2.457432713
sp|P28288|ABCD3_HUMAN	2.427770304
sp|Q9NX05|F120C_HUMAN	2.399710518
sp|A6NJ78|MET15_HUMAN	2.374504793
sp|O75691|UTP20_HUMAN	2.374504793
sp|B9ZVM9|TCP2L_HUMAN	2.349157837
sp|Q9Y262|EIF3L_HUMAN	2.349157837
sp|Q8N1N4|K2C78_HUMAN	2.328583113
sp|O00268|TAF4_HUMAN	2.328583113
sp|Q93100|KPBB_HUMAN	2.328583113
sp|O15457|MSH4_HUMAN	2.328583113
sp|Q15293|RCN1_HUMAN	2.328583113
sp|O43772|MCAT_HUMAN	2.293160547
sp|Q15323|K1H1_HUMAN	2.293160547
sp|Q9H7H0|MET17_HUMAN	2.293160547
sp|P07737|PROF1_HUMAN	2.293160547
sp|O95235|KI20A_HUMAN	2.293160547
sp|Q15059|BRD3_HUMAN	2.293160547
sp|Q9NSI2|F207A_HUMAN	2.278946046
sp|Q2TB18|ASTE1_HUMAN	2.278946046
sp|Q4KMX7|F106A_HUMAN	2.278946046
sp|Q9NZV5|SELN_HUMAN	2.278946046
sp|Q92621|NU205_HUMAN	2.278946046
sp|Q9P2K5|MYEF2_HUMAN	2.278946046
sp|Q08188|TGM3_HUMAN	2.240704963
sp|Q969N2|PIGT_HUMAN	2.224343963
sp|Q96QT4|TRPM7_HUMAN	2.224343963
sp|O75146|HIP1R_HUMAN	2.210022104
sp|Q8N302|AGGF1_HUMAN	2.210022104

sp|P78549|NTH_HUMAN	2.210022104
sp|Q08AD1|CAMP2_HUMAN	2.193248613
sp|Q8WU08|ST32A_HUMAN	2.162337321
sp|P34897|GLYM_HUMAN	2.14733119
sp|O95747|OXSR1_HUMAN	2.135296958
sp|P30876|RPB2_HUMAN	2.135296958
sp|Q9Y6K5|OAS3_HUMAN	2.135296958
sp|Q14134|TRI29_HUMAN	2.135296958
sp|Q15048|LRC14_HUMAN	2.122438263
sp|Q9P015|RM15_HUMAN	2.096354678
sp|P05362|ICAM1_HUMAN	2.085775023
sp|Q14764|MVP_HUMAN	2.085775023
sp|O60884|DNJA2_HUMAN	2.075555496
sp|Q9Y2Q0|AT8A1_HUMAN	2.075555496
sp|Q15286|RAB35_HUMAN	2.075555496
sp|O95158|NXPH4_HUMAN	2.064406781
sp|Q13454|TUSC3_HUMAN	2.031099013
sp|Q6WRI0|IGS10_HUMAN	2.031099013
sp|Q96JN8|NEUL4_HUMAN	2.031099013
sp|P62070|RRAS2_HUMAN	2.013775454

Table S6. mass spectrometry data of the antisense group (control group)

Protein_ID	Protein_Qscore
sp|P21127|CD11B_HUMAN	86.32313305
sp|P05455|LA_HUMAN	43.14988906
sp|P31942|HNRH3_HUMAN	41.31510338
sp|Q2VIR3|IF2GL_HUMAN	35.80197568
sp|P05198|IF2A_HUMAN	34.08405456
sp|Q12849|GRSF1_HUMAN	33.57113784
sp|Q96QR8|PURB_HUMAN	28.34354116
sp|O95639|CPSF4_HUMAN	27.19627136
sp|Q9UN86|G3BP2_HUMAN	26.44024885
sp|P46060|RAGP1_HUMAN	23.5313867
sp|Q9BT17|MTG1_HUMAN	22.29278412
sp|Q6ZRV2|FA83H_HUMAN	21.1751103
sp|Q00577|PURA_HUMAN	19.78274325
sp|O43791|SPOP_HUMAN	19.35089227
sp|P46777|RL5_HUMAN	17.78371668
sp|Q9Y6A4|CFA20_HUMAN	17.78371668
sp|O95232|LC7L3_HUMAN	17.34898265
sp|Q9Y4C8|RBM19_HUMAN	17.16095509
sp|Q8IVT2|MISP_HUMAN	16.90274937
sp|P06396|GELS_HUMAN	16.3040645
sp|Q13310|PABP4_HUMAN	15.42666752
sp|P40429|RL13A_HUMAN	15.31431423
sp|P15311|EZRI_HUMAN	14.99895847
sp|Q14444|CAPR1_HUMAN	14.7811767
sp|P55209|NP1L1_HUMAN	14.11883202
sp|P52907|CAZA1_HUMAN	14.11883202
sp|Q9UKJ3|GPTC8_HUMAN	14.11883202
sp|Q13151|ROA0_HUMAN	14.11883202
sp|Q8N3Z3|GTPB8_HUMAN	13.23451954
sp|Q5T9A4|ATD3B_HUMAN	13.07743934
sp|Q96AG4|LRC59_HUMAN	13.07743934
sp|Q9BYG3|MK67I_HUMAN	13.07743934
sp|O75909|CCNK_HUMAN	13.07743934
sp|Q13243|SRSF5_HUMAN	12.80317555
sp|O95573|ACSL3_HUMAN	12.80317555
sp|P46087|NOP2_HUMAN	12.32141683
sp|P62995|TRA2B_HUMAN	12.15561424
sp|Q9Y324|FCF1_HUMAN	12.11850392
sp|Q9UQB8|BAIP2_HUMAN	11.11422156
sp|Q96DV4|RM38_HUMAN	10.92835995
sp|Q08170|SRSF4_HUMAN	10.7651413
sp|O00422|SAP18_HUMAN	9.737739238

sp|Q9BRJ7|TIRR_HUMAN	9.705556224
sp|Q04837|SSBP_HUMAN	9.41255468
sp|P84090|ERH_HUMAN	9.41255468
sp|Q9Y4W6|AFG32_HUMAN	9.41255468
sp|P51116|FXR2_HUMAN	9.41255468
sp|Q5T3I0|GPTC4_HUMAN	9.41255468
sp|Q6NZI2|CAVN1_HUMAN	9.41255468
sp|Q9NR12|PDLI7_HUMAN	9.41255468
sp|O95400|CD2B2_HUMAN	9.41255468
sp|P61221|ABCE1_HUMAN	9.41255468
sp|Q9UBB4|ATX10_HUMAN	9.41255468
sp|P62910|RL32_HUMAN	9.41255468
sp|P27824|CALX_HUMAN	9.41255468
sp|P62314|SMD1_HUMAN	8.371161995
sp|Q13148|TADBP_HUMAN	8.371161995
sp|Q3ZCQ8|TIM50_HUMAN	8.371161995
sp|Q02543|RL18A_HUMAN	8.371161995
sp|Q07020|RL18_HUMAN	8.371161995
sp|Q8IXI1|MIRO2_HUMAN	8.371161995
sp|P18085|ARF4_HUMAN	8.371161995
sp|Q9UNX3|RL26L_HUMAN	8.314045525
sp|P46778|RL21_HUMAN	7.9329025
sp|O00541|PESC_HUMAN	7.9329025
sp|P62714|PP2AB_HUMAN	7.748400415
sp|Q9UNF1|MAGD2_HUMAN	7.748400415
sp|P07205|PGK2_HUMAN	7.748400415
sp|Q99623|PHB2_HUMAN	7.748400415
sp|Q8N1G2|CMTR1_HUMAN	7.552998639
sp|Q8NEY8|PPHLN_HUMAN	7.32976931
sp|Q58FF3|ENPLL_HUMAN	7.328065499
sp|Q15007|FL2D_HUMAN	7.241406976
sp|P41250|GARS_HUMAN	7.147294567
sp|Q15397|PUM3_HUMAN	6.951304292
sp|O95373|IPO7_HUMAN	6.887872202
sp|Q96T58|MINT_HUMAN	6.407944216
sp|Q9Y520|PRC2C_HUMAN	5.831638097
sp|Q14103|HNRPD_HUMAN	5.773837725
sp|P68871|HBB_HUMAN	5.719664715
sp|P48643|TCPE_HUMAN	5.392337509
sp|Q15020|SART3_HUMAN	5.021538116
sp|Q9NWB6|ARGL1_HUMAN	5.014745222
sp|P62333|PRS10_HUMAN	4.836243595
sp|P0DOX7|IGK_HUMAN	4.70627734
sp|P62861|RS30_HUMAN	4.70627734

sp|Q9BTD8|RBM42_HUMAN	4.70627734
sp|Q9Y2R9|RT07_HUMAN	4.70627734
sp|Q9UHF1|EGFL7_HUMAN	4.70627734
sp|Q9UPT9|UBP22_HUMAN	4.70627734
sp|P07951|TPM2_HUMAN	4.70627734
sp|P35232|PHB_HUMAN	4.70627734
sp|P08133|ANXA6_HUMAN	4.70627734
sp|Q8WTT2|NOC3L_HUMAN	4.70627734
sp|O75874|IDHC_HUMAN	4.70627734
sp|P35637|FUS_HUMAN	4.70627734
sp|Q7RTY9|PRS41_HUMAN	4.70627734
sp|Q05639|EF1A2_HUMAN	4.70627734
sp|O75369|FLNB_HUMAN	4.70627734
sp|Q86XK2|FBX11_HUMAN	4.70627734
sp|Q14694|UBP10_HUMAN	4.70627734
sp|P62847|RS24_HUMAN	4.70627734
sp|Q5SSJ5|HP1B3_HUMAN	4.70627734
sp|Q9BXK1|KLF16_HUMAN	4.70627734
sp|Q9NXV6|CARF_HUMAN	4.70627734
sp|Q8WXF1|PSPC1_HUMAN	4.70627734
sp|O43896|KIF1C_HUMAN	4.70627734
sp|P41223|BUD31_HUMAN	4.70627734
sp|P01876|IGHA1_HUMAN	4.70627734
sp|Q9BPW8|NIPS1_HUMAN	4.70627734
sp|Q86WX3|AROS_HUMAN	4.70627734
sp|Q9BYJ9|YTHD1_HUMAN	4.70627734
sp|Q53GQ0|DHB12_HUMAN	4.70627734
sp|Q9BRT6|LLPH_HUMAN	4.70627734
sp|O95391|SLU7_HUMAN	4.70627734
sp|O00116|ADAS_HUMAN	4.70627734
sp|O00411|RPOM_HUMAN	4.70627734
sp|Q96A35|RM24_HUMAN	4.70627734
sp|Q9BRX9|WDR83_HUMAN	4.70627734
sp|P19012|K1C15_HUMAN	4.70627734
sp|P08727|K1C19_HUMAN	4.70627734
sp|Q8WXA9|SREK1_HUMAN	4.70627734
sp|Q9ULR0|ISY1_HUMAN	4.70627734
sp|P51114|FXR1_HUMAN	4.70627734
sp|Q99569|PKP4_HUMAN	4.70627734
sp|P09012|SNRPA_HUMAN	4.70627734
sp|Q86V81|THOC4_HUMAN	4.70627734
sp|P56545|CTBP2_HUMAN	4.70627734
sp|Q9UPN6|SCAF8_HUMAN	4.70627734
sp|Q15021|CND1_HUMAN	4.70627734

sp|P13984|T2FB_HUMAN	4.70627734
sp|P30041|PRDX6_HUMAN	4.70627734
sp|Q13867|BLMH_HUMAN	4.70627734
sp|Q9H4L4|SENP3_HUMAN	4.70627734
sp|P16070|CD44_HUMAN	4.70627734
sp|Q8N9T8|KRI1_HUMAN	4.70627734
sp|P36873|PP1G_HUMAN	4.70627734
sp|Q99959|PKP2_HUMAN	4.70627734
sp|P61313|RL15_HUMAN	4.70627734
sp|O96008|TOM40_HUMAN	4.70627734
sp|Q13509|TBB3_HUMAN	4.70627734
sp|Q3ZCM7|TBB8_HUMAN	4.70627734
sp|Q6UXN9|WDR82_HUMAN	4.70627734
sp|Q9BW27|NUP85_HUMAN	4.70627734
sp|P23378|GCSP_HUMAN	4.70627734
sp|P62888|RL30_HUMAN	4.70627734
sp|Q969S9|RRF2M_HUMAN	4.70627734
sp|P13804|ETFA_HUMAN	4.70627734
sp|P00338|LDHA_HUMAN	4.70627734
sp|P19367|HXK1_HUMAN	4.70627734
sp|Q9BQE3|TBA1C_HUMAN	4.70627734
sp|O15235|RT12_HUMAN	4.70627734
sp|Q9UH17|ABC3B_HUMAN	4.70627734
sp|P07814|SYEP_HUMAN	4.70627734
sp|Q7Z7H8|RM10_HUMAN	4.70627734
sp|P82650|RT22_HUMAN	4.70627734
sp|P14373|TRI27_HUMAN	4.70627734
sp|Q9UHB6|LIMA1_HUMAN	4.70627734
sp|P53985|MOT1_HUMAN	4.70627734
sp|P22392|NDKB_HUMAN	4.70627734
sp|P50990|TCPQ_HUMAN	4.70627734
sp|P23921|RIR1_HUMAN	4.70627734
sp|Q96S52|PIGS_HUMAN	4.70627734
sp|P07858|CATB_HUMAN	4.70627734
sp|Q12788|TBL3_HUMAN	4.70627734
sp|P49755|TMEDA_HUMAN	4.70627734
sp|Q6DCA0|AMERL_HUMAN	4.70627734
sp|P35659|DEK_HUMAN	3.664884655
sp|Q12797|ASPH_HUMAN	3.664884655
sp|Q96LU7|MRFL_HUMAN	3.664884655
sp|Q96N67|DOCK7_HUMAN	3.664884655
sp|P62136|PP1A_HUMAN	3.664884655
sp|A0AV96|RBM47_HUMAN	3.664884655
sp|P78362|SRPK2_HUMAN	3.664884655

sp|P09874|PARP1_HUMAN	3.664884655
sp|Q9Y3C6|PPIL1_HUMAN	3.664884655
sp|P18669|PGAM1_HUMAN	3.664884655
sp|Q9Y5Y2|NUBP2_HUMAN	3.664884655
sp|Q92522|H1X_HUMAN	3.664884655
sp|Q03701|CEBPZ_HUMAN	3.664884655
sp|Q8IXT5|RB12B_HUMAN	3.664884655
sp|O43432|IF4G3_HUMAN	3.664884655
sp|Q9NQ29|LUC7L_HUMAN	3.664884655
sp|Q969Q0|RL36L_HUMAN	3.664884655
sp|P13929|ENOB_HUMAN	3.664884655
sp|Q9NRK6|ABCBA_HUMAN	3.664884655
sp|Q92552|RT27_HUMAN	3.664884655
sp|Q92759|TF2H4_HUMAN	3.664884655
sp|Q96A72|MGN2_HUMAN	3.664884655
sp|Q9UQ16|DYN3_HUMAN	3.664884655
sp|P84085|ARF5_HUMAN	3.664884655
sp|Q9BWJ5|SF3B5_HUMAN	3.664884655
sp|Q16637|SMN_HUMAN	3.664884655
sp|Q9BVP2|GNL3_HUMAN	3.390620871
sp|Q9Y2T2|AP3M1_HUMAN	3.390620871
sp|Q15646|OASL_HUMAN	3.390620871
sp|O43776|SYNC_HUMAN	3.390620871
sp|Q13162|PRDX4_HUMAN	3.390620871
sp|P60228|EIF3E_HUMAN	3.22662516
sp|P48444|COPD_HUMAN	3.22662516
sp|Q71RC2|LARP4_HUMAN	3.22662516
sp|Q96QV6|H2A1A_HUMAN	3.22662516
sp|Q9Y3Q3|TMED3_HUMAN	3.22662516
sp|Q9BW92|SYTM_HUMAN	3.22662516
sp|O00217|NDUS8_HUMAN	3.108144158
sp|P54098|DPOG1_HUMAN	3.108144158
sp|O75131|CPNE3_HUMAN	3.108144158
sp|Q96HE7|ERO1A_HUMAN	3.042123074
sp|Q14554|PDIA5_HUMAN	3.042123074
sp|P35749|MYH11_HUMAN	3.042123074
sp|P49821|NDUV1_HUMAN	3.042123074
sp|Q9UQE7|SMC3_HUMAN	3.042123074
sp|Q9UBM7|DHCR7_HUMAN	3.042123074
sp|P48735|IDHP_HUMAN	3.042123074
sp|Q15058|KIF14_HUMAN	3.042123074
sp|P10321|HLAC_HUMAN	3.042123074
sp|Q9Y6B6|SAR1B_HUMAN	3.042123074
sp|Q96NC0|ZMAT2_HUMAN	3.042123074

sp|Q9NY61|AATF_HUMAN	3.042123074
sp|Q12894|IFRD2_HUMAN	3.042123074
sp|P09001|RM03_HUMAN	3.042123074
sp|Q7Z7B0|FLIP1_HUMAN	3.042123074
sp|Q5SW79|CE170_HUMAN	3.042123074
sp|P55735|SEC13_HUMAN	2.965641788
sp|O95466|FMNL1_HUMAN	2.965641788
sp|P51553|IDH3G_HUMAN	2.908862149
sp|Q9H936|GHC1_HUMAN	2.908862149
sp|Q13459|MYO9B_HUMAN	2.908862149
sp|P51649|SSDH_HUMAN	2.908862149
sp|O75390|CISY_HUMAN	2.908862149
sp|Q8WUQ7|CATIN_HUMAN	2.858034303
sp|Q96DH6|MSI2H_HUMAN	2.858034303
sp|Q8IYJ2|CJ067_HUMAN	2.858034303
sp|O00299|CLIC1_HUMAN	2.858034303
sp|Q6P1K8|T2H2L_HUMAN	2.858034303
sp|O60333|KIF1B_HUMAN	2.81142407
sp|P56134|ATPK_HUMAN	2.76818487
sp|Q69YN2|C19L1_HUMAN	2.76818487
sp|Q13347|EIF3I_HUMAN	2.76818487
sp|P08134|RHOC_HUMAN	2.743059561
sp|Q8IUG5|MY18B_HUMAN	2.743059561
sp|Q969G5|CAVN3_HUMAN	2.743059561
sp|Q5SNV9|CA167_HUMAN	2.743059561
sp|Q5VWN6|TASO2_HUMAN	2.743059561
sp|O76013|KRT36_HUMAN	2.743059561
sp|O75934|SPF27_HUMAN	2.743059561
sp|Q5T8P6|RBM26_HUMAN	2.67754164
sp|Q16658|FSCN1_HUMAN	2.67754164
sp|Q96MU7|YTDC1_HUMAN	2.67754164
sp|Q9Y2P8|RCL1_HUMAN	2.67754164
sp|P82914|RT15_HUMAN	2.67754164
sp|O15488|GLYG2_HUMAN	2.67754164
sp|Q9BZE1|RM37_HUMAN	2.67754164
sp|P36507|MP2K2_HUMAN	2.67754164
sp|Q96LT9|RNPC3_HUMAN	2.67754164
sp|O14617|AP3D1_HUMAN	2.649277948
sp|H7C350|CC188_HUMAN	2.649277948
sp|P02748|CO9_HUMAN	2.621788159
sp|Q96S94|CCNL2_HUMAN	2.621788159
sp|P25440|BRD2_HUMAN	2.596926464
sp|P40227|TCPZ_HUMAN	2.596926464
sp|Q14684|RRP1B_HUMAN	2.574631068

sp|Q9NQ94|A1CF_HUMAN	2.574631068
sp|O15427|MOT4_HUMAN	2.574631068
sp|Q14254|FLOT2_HUMAN	2.557197954
sp|Q4AC99|1A1L2_HUMAN	2.557197954
sp|Q96QA5|GSDMA_HUMAN	2.557197954
sp|Q15050|RRS1_HUMAN	2.557197954
sp|Q96EY4|TMA16_HUMAN	2.557197954
sp|P04075|ALDOA_HUMAN	2.557197954
sp|Q9BRR6|ADPGK_HUMAN	2.557197954
sp|Q01534|TSPY1_HUMAN	2.557197954
sp|Q92979|NEP1_HUMAN	2.557197954
sp|Q14789|GOGB1_HUMAN	2.557197954
sp|Q8IYW2|CFA46_HUMAN	2.557197954
sp|Q9Y224|RTRAF_HUMAN	2.557197954
sp|O43889|CREB3_HUMAN	2.557197954
sp|O75319|DUS11_HUMAN	2.492566989
sp|A6NK89|RASFA_HUMAN	2.474263945
sp|Q9UKN1|MUC12_HUMAN	2.474263945
sp|O15061|SYNEM_HUMAN	2.474263945
sp|O43823|AKAP8_HUMAN	2.457547268
sp|Q5SRN2|TSBP1_HUMAN	2.457547268
sp|Q6P3W6|NBPFA_HUMAN	2.441017227
sp|Q7KZF4|SND1_HUMAN	2.441017227
sp|O60341|KDM1A_HUMAN	2.441017227
sp|Q96BD5|PF21A_HUMAN	2.424611652
sp|Q8TDZ2|MICA1_HUMAN	2.409066113
sp|Q9H8Y5|ANKZ1_HUMAN	2.350000935
sp|Q8N954|GPT11_HUMAN	2.337729957
sp|Q9HB29|ILRL2_HUMAN	2.337729957
sp|P46779|RL28_HUMAN	2.337729957
sp|P38606|VATA_HUMAN	2.32512359
sp|Q8NFI4|F10A5_HUMAN	2.287051083
sp|Q96JN2|CC136_HUMAN	2.276067908
sp|Q6IEU7|OR5MA_HUMAN	2.276067908
sp|P07195|LDHB_HUMAN	2.276067908
sp|Q14571|ITPR2_HUMAN	2.265157279
sp|Q9H1A4|APC1_HUMAN	2.265157279
sp|P62308|RUXG_HUMAN	2.245026952
sp|Q7Z4Q2|HEAT3_HUMAN	2.245026952
sp|Q5U651|RAIN_HUMAN	2.245026952
sp|Q96SI9|STRBP_HUMAN	2.236874898
sp|Q86X95|CIR1_HUMAN	2.236874898
sp|Q9Y6H5|SNCAP_HUMAN	2.236874898
sp|Q9H773|DCTP1_HUMAN	2.236874898

sp|O60313|OPA1_HUMAN	2.236874898
sp|Q8NEN0|ARMC2_HUMAN	2.236874898
sp|Q8TDN4|CABL1_HUMAN	2.226526133
sp|O60346|PHLP1_HUMAN	2.216264578
sp|Q92673|SORL_HUMAN	2.189937179
sp|P49137|MAPK2_HUMAN	2.181594862
sp|P12814|ACTN1_HUMAN	2.181594862
sp|P55039|DRG2_HUMAN	2.181594862
sp|Q9HBH1|DEFM_HUMAN	2.181594862
sp|Q9NTX5|ECHD1_HUMAN	2.181594862
sp|Q13617|CUL2_HUMAN	2.164754329
sp|O43615|TIM44_HUMAN	2.156241881
sp|Q9BQA1|MEP50_HUMAN	2.148159335
sp|O43447|PPIH_HUMAN	2.139665809
sp|Q92688|AN32B_HUMAN	2.10895307
sp|Q9NW38|FANCL_HUMAN	2.10895307
sp|P07384|CAN1_HUMAN	2.10895307
sp|Q5XPI4|RN123_HUMAN	2.10895307
sp|P06241|FYN_HUMAN	2.101809994
sp|Q12834|CDC20_HUMAN	2.101809994
sp|P41219|PERI_HUMAN	2.101809994
sp|P17181|INAR1_HUMAN	2.046017841
sp|Q9HBE1|PATZ1_HUMAN	2.027482144
sp|O75323|NIPS2_HUMAN	2.027482144
sp|Q96EY1|DNJA3_HUMAN	2.016047042
sp|Q9NYQ8|FAT2_HUMAN	2.016047042
sp|Q3B726|RPA43_HUMAN	2.010138052
